# Machine Learning to Assist in Managing Acute Kidney Injury in General Wards: Multicenter Retrospective Study

**DOI:** 10.2196/66568

**Published:** 2025-03-18

**Authors:** Nam-Jun Cho, Inyong Jeong, Se-Jin Ahn, Hyo-Wook Gil, Yeongmin Kim, Jin-Hyun Park, Sanghee Kang, Hwamin Lee

**Affiliations:** 1 Department of Internal Medicine Soonchunhyang University Cheonan Hospital Cheonan Republic of Korea; 2 Department of Biomedical Informatics Korea University College of Medicine Seoul Republic of Korea; 3 Department of Surgery Korea University Guro Hospital Seoul Republic of Korea

**Keywords:** acute kidney injury, machine learning, recovery of function, creatinine, kidney, patient rooms

## Abstract

**Background:**

Most artificial intelligence–based research on acute kidney injury (AKI) prediction has focused on intensive care unit settings, limiting their generalizability to general wards. The lack of standardized AKI definitions and reliance on intensive care units further hinder the clinical applicability of these models.

**Objective:**

This study aims to develop and validate a machine learning–based framework to assist in managing AKI and acute kidney disease (AKD) in general ward patients, using a refined operational definition of AKI to improve predictive performance and clinical relevance.

**Methods:**

This retrospective multicenter cohort study analyzed electronic health record data from 3 hospitals in South Korea. AKI and AKD were defined using a refined version of the Kidney Disease: Improving Global Outcomes criteria, which included adjustments to baseline serum creatinine estimation and a stricter minimum increase threshold to reduce misclassification due to transient fluctuations. The primary outcome was the development of machine learning models for early prediction of AKI (within 3 days before onset) and AKD (nonrecovery within 7 days after AKI).

**Results:**

The final analysis included 135,068 patients. A total of 7658 (8%) patients in the internal cohort and 2898 (7.3%) patients in the external cohort developed AKI. Among the 5429 patients in the internal cohort and 1998 patients in the external cohort for whom AKD progression could be assessed, 896 (16.5%) patients and 287 (14.4%) patients, respectively, progressed to AKD. Using the refined criteria, 2898 cases of AKI were identified, whereas applying the standard Kidney Disease: Improving Global Outcomes criteria resulted in the identification of 5407 cases. Among the 2509 patients who were not classified as having AKI under the refined criteria, 2242 had a baseline serum creatinine level below 0.6 mg/dL, while the remaining 267 experienced a decrease in serum creatinine before the onset of AKI. The final selected early prediction model for AKI achieved an area under the receiver operating characteristic curve of 0.9053 in the internal cohort and 0.8860 in the external cohort. The early prediction model for AKD achieved an area under the receiver operating characteristic curve of 0.8202 in the internal cohort and 0.7833 in the external cohort.

**Conclusions:**

The proposed machine learning framework successfully predicted AKI and AKD in general ward patients with high accuracy. The refined AKI definition significantly reduced the classification of patients with transient serum creatinine fluctuations as AKI cases compared to the previous criteria. These findings suggest that integrating this machine learning framework into hospital workflows could enable earlier interventions, optimize resource allocation, and improve patient outcomes.

## Introduction

Acute kidney injury (AKI) is an escalating critical health and socioeconomic issue, marked by prolonged hospital stays, elevated medical costs, and high mortality rates [1]. AKI is a secondary condition arising from hospital interventions, such as medication, surgery, and infections, with a prevalence of 10%-15% among general inpatients [2]. Studies have shown that the duration of AKI correlates with increased risks of complications and mortality [3]. AKI and its progression to acute kidney disease (AKD) are associated with significant increases in postdischarge morbidity and mortality. The prolonged duration of AKI in general wards has been associated with higher risks of complications and increased mortality [3-5]. Early prediction of AKI and its progression to AKD using artificial intelligence (AI) models improve patient outcomes through timely intervention and personalized management strategies [5-7]. There are research findings indicating that applying a clinical decision support system for actual AKI occurrences has significantly improved patient outcomes [8-10]. However, there is a lack of standardized operational definitions for AKI, particularly in retrospective studies, where data collection may not be systematic [11,12]. This inconsistency in defining AKI, especially in terms of baseline serum creatinine (SCr) and recovery criteria for AKD, leads to ambiguity in labeling and makes it difficult to compare the performance of different AI models across studies [13,14]. The variability in baseline SCr determination further complicates the issue [14-16]. Studies that investigated the impact of baseline SCr on model performance are limited to the intensive care unit (ICU) setting and do not address real-time early prediction in general wards [17-19], in differing AKI labels for the same patient. Defining the recovery for AKD is even more challenging [4,20-23].

General ward patients typically exhibit milder disease severity, less frequent laboratory measurements, and different baseline characteristics compared with ICU patients [15,20,24-27]. Applying the criterion of baseline SCr being 1.5 times higher requires more caution, especially in cases with low SCr levels. For instance, in a patient with 3 SCr measurements within 48 hours showing values of 0.9 mg/dL, 0.6 mg/dL, and 0.9 mg/dL in chronological order, additional consideration may be needed to determine whether the patient should be classified as experiencing AKI. Furthermore, when the baseline SCr is set below 0.6 mg/dL, even small fluctuations can easily meet the 1.5-fold criterion within 7 days, leading to the classification of AKI occurrence [28]. This study aimed to develop and validate a machine learning-based framework for early prediction of AKI and AKD, applicable to general ward patients, using a refined operational definition of AKI.

## Methods

### Study Setting and Participants

This multicenter, retrospective study was conducted across the general wards of 3 different hospitals. Data were collected from patients admitted to Korea University Guro Hospital and Anam Hospital between January 1, 2015, and December 31, 2021 (internal cohort), and from Soonchunhyang University Cheonan Hospital between March 1, 2016, and March 31, 2021 (external cohort). Patients younger than 19 years, those with fewer than 3 SCr measurements during their hospital stay, and those with an estimated glomerular filtration rate (eGFR) ≤60 mL/minute on the first day of SCr measurement were excluded.

### Operative Definition

AKI was defined based on the Kidney Disease: Improving Global Outcomes (KDIGO) criteria as follows [1].

- An increase in SCr level ≥0.3 mg/dL (or ≥26.5 μmol/L) within 48 h.
- An increase in SCr ≥1.5 times the baseline within 7 days.

The KDIGO criteria are widely used; however, they are sometimes modified based on the characteristics of the cohort or the objectives of the study [28]. Compared with ICU patients, general ward patients are in relatively better condition with less frequent measurements of vital signs and laboratory data. Additionally, individuals with healthy kidneys and low SCr levels can be identified as having AKI due to simple fluctuations [14]. There may be more bias in real-time predictions, as SCr estimation is more frequently performed for patients with missing SCr values. Therefore, to prevent these issues, we restricted the application of baseline SCr levels and refined the labeling. To improve the labeling criteria, baseline SCr was defined as the lowest SCr measured within the previous 7 days. If the patient’s baseline SCr was ≥0.3 mg/dL lower than the median and recent SCr values, it was excluded as a baseline to avoid errors due to SCr measurement inaccuracies or temporary fluctuations caused by medication, measurement error, and other factors. AKI was defined as a minimum increase of 0.3 mg/dL to ensure appropriate identification without mislabeling due to minor variations [2,29,30]. AKD was defined as the persistence of AKI for more than 7 days. Cases in which SCr did not return to <1.5 times the baseline SCr within 7 days were identified as AKD [21,29-32]. None of the previous definitions or studies have taken these aspects into consideration.

To develop an early prediction model for AKI occurrence, data from 1 to 3 days before AKI onset were labeled as 1, and the remaining data were labeled as 0. The data from the day of AKI onset were not used. For patients who did not develop AKI, all data were labeled as 0. Days without SCr measurements within 7 days were excluded from training and evaluation. Additionally, patients with ambiguous AKI labeling were excluded from the training and evaluation. Data from the day of AKI onset were used to predict AKD progression. Patients were labeled 0 or 1 based on whether they recovered within 7 days post AKI onset. Patients with insufficient SCr measurements after AKI onset were excluded. Multimedia Appendix 1 illustrates the previous KDIGO criteria, the improved AKI criteria proposed in this study, and examples of AKI and AKD labeling.

### Data Preprocessing

The data collected from the electronic health records contained numerous missing values, including measurement values, times, and specific variables. To address this issue, data were summarized at 24-hour intervals, a method validated in previous studies [33]. Vital signs, laboratory data, and variables such as nephrotoxic drugs (eg, nonsteroidal anti-inflammatory drugs, nephrotoxic antibiotics [ANTIs], and cytotoxic chemotherapeutic agents), vascular imaging examinations, surgeries under general anesthesia, contrast-enhanced computed tomography (CECT), and ICU transfers within the past 7 days were collected.

Vital sign data measured multiple times within 24 hours were summarized using mean, maximum, and minimum values, and the number of measurements. The laboratory test results were determined based on recent measurements. Additionally, variables such as nephrotoxic drugs, vascular imaging examinations, surgeries under general anesthesia, CECT, and ICU transfers within the past 7 days were included. eGFR was calculated using the Chronic Kidney Disease Epidemiology Collaboration 2021 equation [34]. The “BUN/Cr ratio” was defined as blood urea nitrogen (BUN) level divided by serum creatinine level.

Robust scaling was applied to all continuous variables and one-hot encoding was used for categorical variables. Approximately 120 features were extracted from the electronic health records, including basic patient information, vital sign data, laboratory test results, and other factors. Through a comprehensive literature review and consultation with specialists, a feature selection process was undertaken [6,23,35], and 42 features were ultimately selected based on their correlation coefficients and missing value ratios.

To handle outliers, the data distribution for each feature was reviewed along with the individual patient records. Outliers for some numerical variables were determined using histograms and quantiles with input from clinical experts. The missing values were imputed in 2 stages. First, where feasible, missing values were replaced with previous values to maintain data continuity. For variables with low missing values (less than 20%), the multiple imputation by chained equations method was used [36,37]. For variables with a missing rate exceeding 20%, the missing indicator method was used to denote missing values as unknown [38]. Numerical variables were categorized into 2 to 4 groups based on data distribution and expert consultation, and missing values were assigned to the “missing” category. For laboratory test results not subjected to missing indicators, changes in each variable were calculated by subtracting the median of the previous values from the current value (Multimedia Appendix 2).

### Model Training and Evaluation

Various traditional machine learning models have been used, including logistic regression [39], random forest [40], eXtreme gradient boosting [41], light gradient boosting machine [42], and categorical boosting (CAT) [43]. For detailed model training procedures (Multimedia Appendix 3). The model evaluation metrics included accuracy, precision, recall, specificity, *F*_1_-score, the area under the receiver operating characteristic (AUROC), and the area under the precision-recall curve. The primary outcomes were the development of models for early AKI prediction within 3 days and its progression to AKD, along with the establishment of a framework. Simulations of the designed framework were repeatedly performed using the external validation cohort by dividing the period into 1-year increments and assessing the generalization performance of the models across different institutions and over time.

### Statistical Analysis

Descriptive statistics were used to present the baseline differences between the days with and without AKI. The distribution of continuous and categorical variables was expressed as means and SDs and counts and percentages, respectively. Normally distributed continuous variables were evaluated using 2-tailed *t* tests. For those that did not follow a normal distribution, the Mann-Whitney *U* test was used. The chi-square test was used to analyze categorical variables. Statistical significance was set at *P*<.05. Calibration plots were used to assess the agreement between the predicted probabilities and observed outcomes [44]. The Cox proportional hazards model was used to compare hazard ratios (HRs) between patients with actual AKD and those predicted by the model [45].

### Ethical Considerations

The study was conducted in accordance with the ethical principles of the Declaration of Helsinki and was approved by the institutional review boards of Soonchunhyang University Cheonan Hospital, Korea University Anam Hospital, and Guro Hospital (approvals 2019-10-023, 2023AN0145, and 2023GR0425, respectively). The need for individual consent was waived due to the retrospective nature of the study and the use of anonymized clinical data. To ensure privacy and data security, only fully anonymized data were used, and all analyses were conducted within a designated secure environment with restricted access. No financial compensation was provided to participants, as the study was purely observational and used deidentified retrospective data. All processes adhered to the guidelines developed for machine learning model development in the biomedical field [46] and followed the STROE (Strengthening the Reporting of Observational Studies in Epidemiology) guidelines for observational studies.

## Results

### Labeling

**Figure 1:**
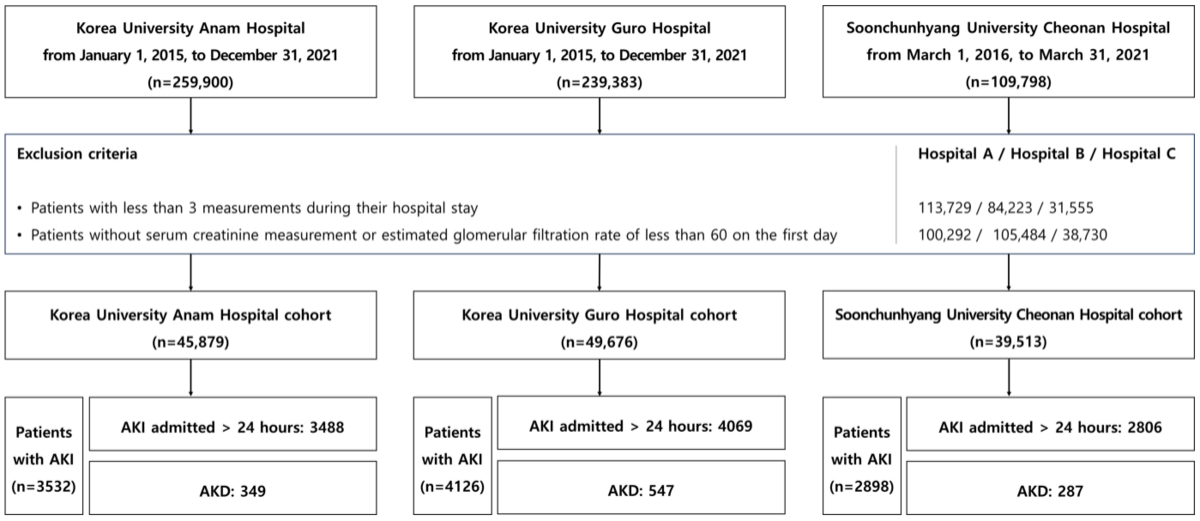
The data composition process. Hospitals A, B, and C refer to Korea University Anam Hospital, Korea University Guro Hospital, and Soonchunhyang University Cheonan Hospital, respectively. Since at least 1 day of data is required for model prediction, patients who developed AKI on the first day of hospitalization were excluded from the final cohort. AKI: acute kidney injury; AKD: acute kidney disease.

**Figure 2:**
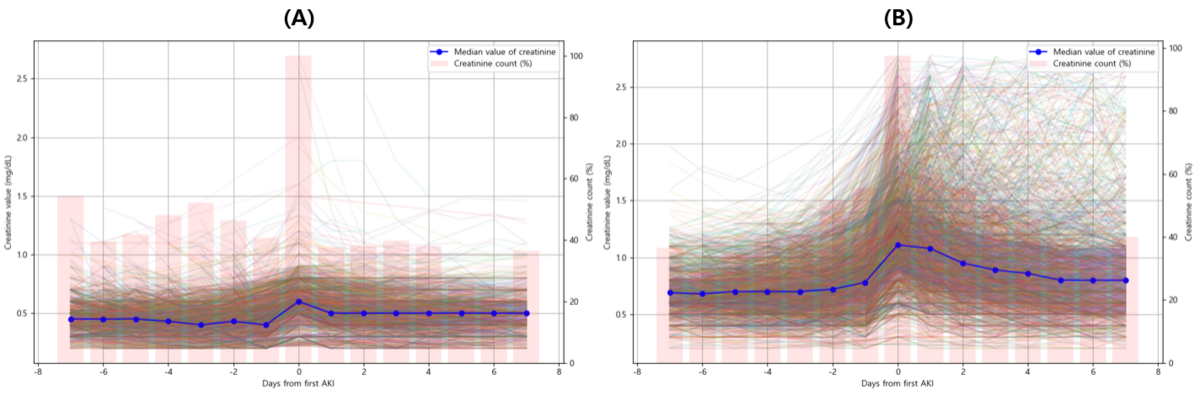
Serum creatinine trends in patients with AKI from the external validation cohort. (A) Results for 2509 patients who meet only the previous AKI criteria. (B) Results for 2898 patients who meet the refined AKI criteria. The blue line represents the median value for patients of each group, while the pink bar graph shows the proportion of serum creatinine measurements across all patients within 7 days before and after the day of acute kidney injury (day 0). The colored lines depict the serum creatinine levels for individual patients. AKI: acute kidney injury.

Figure 1 is a flowchart of cohort formation and labeling. To compare the previous labeling criteria [7,32,47-49] with the labeling criteria used in this study, we developed and evaluated the model using the same methodology. Additionally, we calculated and presented the HRs for a 30% or 40% reduction in eGFR at the time of AKI onset (Figure 2 and Multimedia Appendix 4). In Table S7 in Multimedia Appendix 4, individuals with a baseline SCr<0.6 mg/dL were included, leading to the classification of AKI in patients with very low changes in SCr or very low baseline SCr levels. Additionally, subtracting the baseline median from the SCr at this point yielded a median of 0.10 (IQR 0.00-0.20).

Figure 2A illustrates this trend. Of the 2509 patients, 2242 (89.36%) had a baseline SCr<0.6 mg/dL at the time of AKI occurrence. The remaining 267 patients experienced decreased SCr levels before being diagnosed with AKI. This trend is illustrated in Figure S5 in Multimedia Appendix 4. Additionally, of the 2072 patients whose AKD status could be determined, only 50 (2.41%) progressed to AKD, and only 1 patient experienced a more than 30% decrease in eGFR within 30 days of AKI onset.

### Early AKI Prediction Model

The final analysis included 95,555 and 39,513 cases in the internal and external cohorts, respectively. Within these cohorts, AKI was identified in 7658 (8.1%) and 2898 (7.3%) patients, respectively. The median length of hospital stay was 11 (IQR 7-20) days for the internal cohort and 10 (IQR 6-18) days for the external cohort. The median number of days to AKI occurrence from admission was 8 (IQR 3-17) days for the internal cohort and 7 (IQR 3-16) days for the external cohort (Multimedia Appendix 5). Tables S9 and Table S10 in Multimedia Appendix 5 present the basic statistics of patients with and without AKI. Comparing the days on which AKI occurred to the days on which it did not, there were statistically significant differences in most characteristics between the internal and external cohorts. However, differences in blood pressure and sodium and chloride levels were observed between the internal and external cohorts.

The models were evaluated based on the results of 5-fold cross-validation, with all performance metrics presented at a cutoff of 0.5. The CAT model demonstrated a strong predictive capability (AUROC=0.9134). The logistic regression model performed poorly (AUROC=0.7754). Model evaluation was conducted through both internal and external validations, showing a slight decrease in performance based on the AUROC (range 0.0097-0.0216). Table 1 presents the performance evaluation results of the early AKI prediction model. The hyperparameters used in the model are listed in Table S11 in Multimedia Appendix 5.

**Figure 3:**
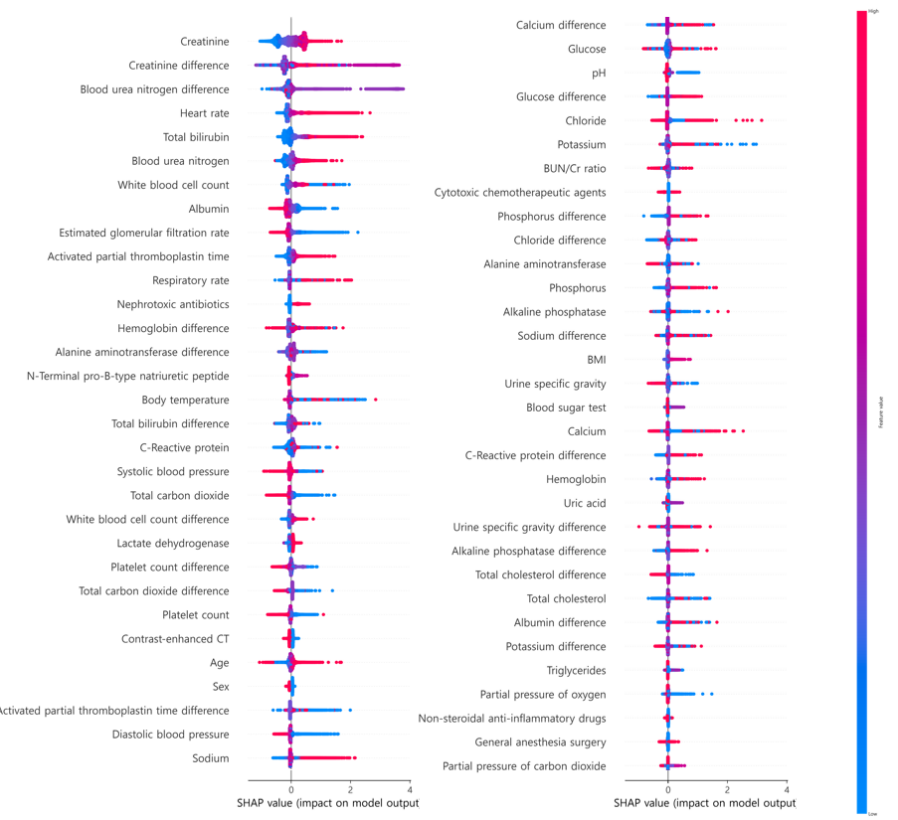
SHAP values of the early prediction model for acute kidney injury. In the figure, red indicates higher values of the respective features, whereas blue indicates lower values. The position of the dots placed towards the right signifies a greater contribution of the feature value to the model’s prediction of nonrecovery for the patient. For categorical variables, red dots represent a value of 1 and blue dots signify a value of 0. CT: computed tomography; SHAP: Shapley additive explanations.

Figure 3 shows the Shapley additive explanations (SHAP) analysis for the early AKI prediction model. The interpretation of the CAT model using SHAP values indicated that the most important features were the SCr level and its changes. Other significant features included heart rate, BUN level, activated partial thromboplastin time (aPTT), total bilirubin level, and BMI, all of which increased the AKI risk. Conversely, lower eGFR, platelet count, body temperature, and pH were associated with a higher risk of AKI. Figure S7 in Multimedia Appendix 5 shows the results of testing the probability of the early AKI prediction model. Calibration plots were generated for both the internal and external cohorts. The slope of the calibration plot was 1.15 for the internal cohort and 1.08 for the external cohort, indicating a good calibration of the model's predicted probabilities. Additionally, the model probabilities were evaluated by dividing them into five groups, which demonstrated excellent calibration. A box plot of the probabilities in relation to the AKI status showed a clear distinction between AKI and non-AKI, further validating the predictive value of the model (Figure S8 in Multimedia Appendix 5).

**Table 1:** Performance evaluation results of the early prediction model for acute kidney injury.

Validation and model		Accuracy	Precision	Recall	*F*_1_-score	AUROC^a^	AUPRC^b^
**Cross-validation, mean (SD)**							
	LR^c^	0.9490 (0.0004)	0.3764 (0.0193)	0.0747 (0.0074)	0.1246 (0.0104)	0.7754 (0.0049)	0.1810 (0.0045)
	RF^d^	0.9739 (0.0004)	0.9875 (0.0020)	0.4687 (0.0091)	0.6356 (0.0080)	0.9076 (0.0060)	0.6830 (0.0076)
	XGB^e^	0.9720 (0.0006)	0.8149 (0.0148)	0.5485 (0.0091)	0.6555 (0.0066)	0.907 (0.0057)	0.6830 (0.0062)
	LGBM^f^	0.9734 (0.0006)	0.8679 (0.0184)	0.5362 (0.0066)	0.6627 (0.0064)	0.9132 (0.0053)	0.6916 (0.0061)
	CAT^g^	0.9747 (0.0005)	0.9423 (0.0095)	0.5115 (0.0093)	0.6630 (0.0078)	0.9134 (0.0053)	0.6924 (0.0059)
**Internal**							
	LR	0.9485	0.3728	0.0709	0.1192	0.7584	0.1712
	RF	0.9736	0.9902	0.4675	0.6352	0.9021	0.6826
	XGB	0.9724	0.8322	0.5490	0.6616	0.9027	0.6834
	LGBM	0.9738	0.8863	0.5348	0.6671	0.9058	0.6910
	CAT	0.9749	0.9527	0.5142	0.6680	0.9053	0.6890
**External**							
	LR	0.9560	0.3612	0.0865	0.1395	0.7487	0.1630
	RF	0.9769	0.9789	0.4502	0.6168	0.8833	0.6303
	XGB	0.9707	0.6938	0.5208	0.5950	0.8811	0.6196
	LGBM	0.9732	0.7651	0.5071	0.6099	0.8853	0.6238
	CAT	0.9755	0.8644	0.4823	0.6191	0.8860	0.6290

^a^AUROC: area under the receiver operating characteristic curve.

^b^AUPRC: area under the precision-recall curve.

^c^LR: logistic regression.

^d^RF: random forest.

^e^XGB: eXtreme gradient boosting.

^f^LGBM: light gradient boosting machine.

^g^CAT: categorical boosting.

### Early AKD Prediction Model

For the analysis of AKD progression, 5429 and 1998 patients in the internal and external cohorts, respectively were included after excluding those with unclear AKD status. Among them, 896 (16.5%) patients in the internal cohort and 287 (14.4%) in the external cohort were diagnosed with AKD. The median duration from AKI to discharge was 10 (IQR 4-23) days in the internal cohort and 9 (IQR 3-21) days in the external cohort. For patients who recovered within 7 days post AKI, the median recovery time was 5 (IQR 3-7) days in both cohorts (Figure S9 in Multimedia Appendix 6). The characteristics of progression to AKD in each cohort are presented in Tables S12 and S13 Multimedia Appendix 6. Variables that demonstrated statistical significance in both cohorts included hemoglobin, albumin, SCr, eGFR, glucose, alanine aminotransferase, blood sugar, calcium, urine specific gravity, C-reactive protein (CRP), ANTIs, and surgery. Some variables such as sex, diastolic blood pressure (DBP), white blood cell (WBC) count, BUN/Cr ratio, and total carbon dioxide showed different trends between the cohorts.

The CAT model outperformed the other models in both the internal (AUROC=0.8202) and external validations (AUROC=0.7833). The external validation showed a slight decrease in performance based on the AUROC (range 0.0262-0.0416). Table 2 presents the performance evaluation results of the early prediction model for AKD. The hyperparameters used in the model are listed in Table S14 in Multimedia Appendix 6.

**Figure 4:**
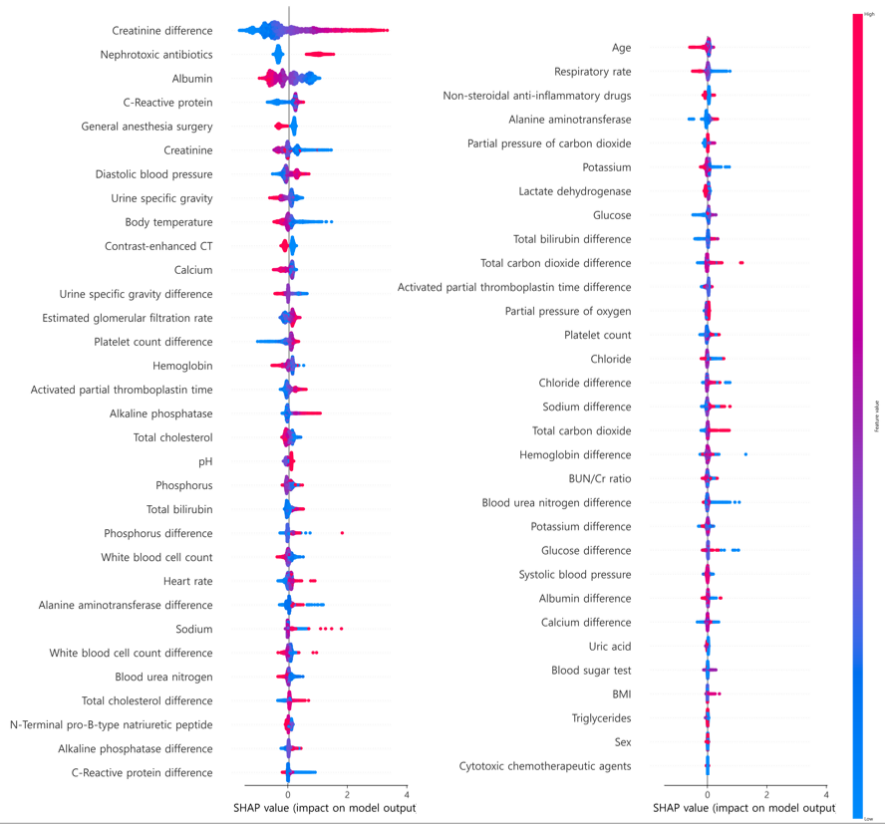
SHAP values of the early prediction model for acute kidney disease. In the figure, red indicates higher values of the respective features, whereas blue indicates lower values. The position of the dots placed towards the right signifies a greater contribution of the feature value to the model's prediction of nonrecovery for the patient. For categorical variables, red dots represent a value of 1 and blue dots signify a value of 0. CT: computed tomography; SHAP: Shapley additive explanations.

Figure 4 shows the SHAP analysis of the early-stage AKD prediction model. For AKD prediction, an increase in SCr level at the time of AKI was a strong predictor of delayed recovery. Exposure to ANTIs, high DBP, CRP, aPTT, low eGFR, low pH, and elevated alkaline phosphatase were associated with an increased risk of AKD. Patients who underwent surgery or CECT tended to recover quicker from AKI.

Figure S10 in Multimedia Appendix 6 shows the results of testing the probability of the early AKD prediction model. Calibration plots for both the internal and external cohorts were generated to assess model performance. The slopes of the calibration plots were 1.27 for the internal cohort and 1.20 for the external cohort, indicating a good calibration of the model’s predicted probabilities. The evaluation of the model probabilities by box plots showed excellent calibration, similar to that of the early prediction model for AKI (Figure S11 in Multimedia Appendix 6).

**Table 2:** Performance evaluation results of the early prediction model for acute kidney disease.

Validation and model		Accuracy	Precision	Recall	*F*_1_-score	AUROC^a^	AUPRC^b^
**Cross-validation, mean (SD)**							
	LR^c^	0.8442 (0.0097)	0.6146 (0.0400)	0.1972 (0.0248)	0.2980 (0.0321)	0.7710 (0.0197)	0.4528 (0.0228)
	RF^d^	0.8370 (0.0089)	0.7711 (0.0728)	0.0432 (0.0066)	0.0819 (0.0123)	0.7702 (0.0158)	0.4346 (0.0166)
	XGB^e^	0.8439 (0.0165)	0.5972 (0.0848)	0.2372 (0.0327)	0.3382 (0.0406)	0.7622 (0.0250)	0.4490 (0.0409)
	LGBM^f^	0.8436 (0.0095)	0.6066 (0.0545)	0.2142 (0.0173)	0.3149 (0.0109)	0.7623 (0.0178)	0.4421 (0.0067)
	CAT^g^	0.8402 (0.0113)	0.6873 (0.0779)	0.0942 (0.0110)	0.1655 (0.0186)	0.7806 (0.0181)	0.4695 (0.0255)
**Internal**							
	LR	0.8460	0.7347	0.2182	0.3364	0.7967	0.5376
	RF	0.8297	0.9000	0.0545	0.1029	0.8099	0.5302
	XGB	0.8547	0.7460	0.2848	0.4123	0.8097	0.5721
	LGBM	0.8557	0.7667	0.2788	0.4089	0.7964	0.5598
	CAT	0.8341	0.8000	0.0970	0.1730	0.8202	0.5558
**External**							
	LR	0.8475	0.5893	0.1019	0.1737	0.7659	0.3959
	RF	0.8446	0.6429	0.0278	0.0533	0.7837	0.4203
	XGB	0.8494	0.5761	0.1636	0.2548	0.7682	0.3990
	LGBM	0.8475	0.5543	0.1574	0.2452	0.7685	0.3945
	CAT	0.8475	0.6786	0.0586	0.1080	0.7833	0.4368

^a^AUROC: area under the receiver operating characteristic curve.

^b^AUPRC: area under the precision-recall curve.

^c^LR: logistic regression.

^d^RF: random forest.

^e^XGB: eXtreme gradient boosting.

^f^LGBM: light gradient boosting machine.

^g^CAT: categorical boosting.

### Framework

**Figure 5:**
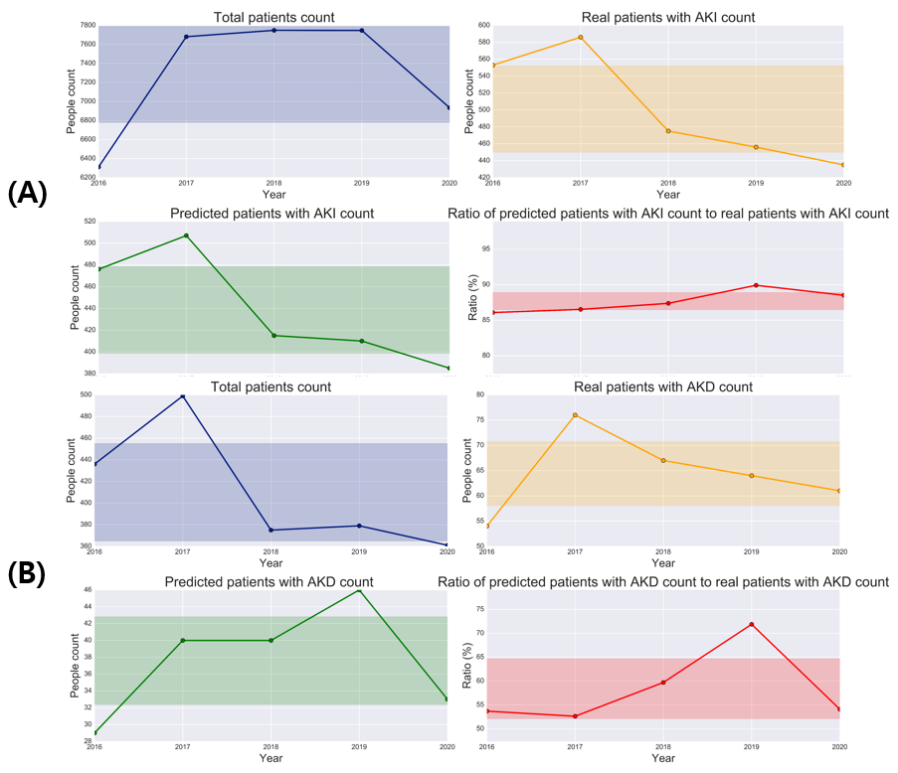
Simulation results of the framework to assist in the management of AKI using external validation data. (A) Results of the early prediction model for AKI, and (B) results of the early prediction model for AKD. Blue indicates patients included in the yearly prediction, orange indicates event occurrence, green indicates patients successfully predicted by the model, and red indicates events successfully predicted by the model compared with actual events. AKI: acute kidney injury; AKD: acute kidney disease.

The framework to assist in managing AKI is shown in Multimedia Appendix 7. Figure 5A presents the framework simulation results for the early AKI prediction model. The designed framework was simulated using an external validation cohort at yearly intervals of 5 years. Among the 7284 patients included annually in the external validation cohort, 501 (6.9%) experienced AKI. Of these, 439 (87.68%) were predicted at least 1 day in advance. The *F*_1_-score showed a decreasing trend after 2018 compared to evaluations between 2016 and 2018, with a minimum of 0.39 and a maximum of 0.46. Figure 5B presents the framework simulation results for the early prediction model for AKD. Excluding patients with insufficient SCr tracking from an average of 410 patients annually, 64 (15.6%) did not recover from AKI within 7 days. Among them, approximately 38 (58.4%) were predicted early.

## Discussion

### Principal Findings

In this study, we developed a framework to assist in the management of AKI. The early AKI prediction model demonstrated high performance with an AUROC of 0.9053, while the early AKD prediction model achieved an AUROC of 0.8202. External validation results showed excellent model performance, although slight variations over time were observed, likely due to changes in disease incidence rates. Our study represents an integrated effort to identify patients with AKI early and, in cases where AKI occurs, classify them into high-risk and low-risk groups. Furthermore, we successfully refined and applied the KDIGO criteria to retrospective data, addressing its limitations and enhancing its applicability for AKI-related research in general ward patients.

Furthermore, our model predicted AKD in patients with AKI and showed an adjusted HR of 2.03 (95% CI 1.38-3.00) for events where eGFR decreased by 30% within 30 days from the onset of AKI. This adjusted HR was higher than that of the model developed using previous criteria, which had an HR of 1.66 (95% CI 1.17-2.34). This indicates that the refined criteria more precisely identify high-risk groups and suggests that the model developed using these criteria performs better. The AKI incidence was higher using the previous criteria, whereas the AKD incidence was higher using the refined criteria. This indicated that the previous criteria identified milder cases. Despite including milder cases, the model developed using the refined criteria showed a higher risk of a poorer prognosis than the previous criteria. Among patients tracked for more than 30 days after AKI, there were 934 and 482 patients before and after criteria advancement, respectively, with 138 and 103 showing poor prognosis. In other words, before the criteria advancement, 35 more patients with a poor prognosis were identified, but 452 additional AKI cases were identified. As shown in Figure S5 in Multimedia Appendix 4, many patients who met only the previous criteria included those whose AKI status was difficult to determine owing to the significant sensitivity to fluctuations. Regardless of how the baseline SCr level is imputed, it is important in general wards to set a minimum increase criterion when applying a relative standard. Additionally, it is crucial to ensure that the baseline SCr level does not become too low when the SCr decreases. Advancements in labeling criteria should consider both patient prognosis and clinical settings. Excessive identification of AKI can lead to the detection of more patients with poor prognoses but may also result in inefficient allocation of medical resources.

Using the SHAP, factors contributing to AKI and AKD were identified and quantitatively measured. These results are largely aligned with the trends suggested by previous studies on risk factors and variable tendencies. Factors indicating poor patient status, such as low albumin or high alkaline phosphatase levels, were associated with increased AKI and AKD risks. Specifically, albumin is considered a critical biomarker closely related to renal function and may decrease in conditions associated with liver dysfunction [50]. Elevated WBC, heart rate, body temperature, and respiratory rate may indicate infection in patients [51], while the use of nephrotoxic drugs such as ANTIs, nonsteroidal anti-inflammatory drugs, and cytotoxic chemotherapeutic agents increases the risk of AKI [52,53]. Analysis of SHAP for AKD risk factors indicated that patients with increased WBC and high BT tended to recover renal function relatively quickly. Resolving infections appears to reduce the risk of AKD development in patients with AKI. ANTIs have emerged as significant risk factors for AKI and AKD. The difficulty in discontinuing these drugs due to patient conditions may exacerbate negative outcomes. Patients undergoing surgery are closely associated with AKI, although AKI following general anesthesia appears to show transient SCr fluctuations and overall health improvement following surgical resolution [54]. Renal function emerged as a crucial factor, consistent with existing clinical studies that state that baseline renal function is well-known as a major factor in AKI [55]. Additionally, higher age, heart rate, respiratory rate, total T-bil, aPTT, and CRP showed increased AKI risk, indicating that poorer patient condition may correlate with increased AKI incidence.

Similarly, poor renal function has been identified as a major risk factor for AKD. Elevated DBP reflects a tendency toward increased blood vessel volume after AKI, suggesting a possible correlation between increased volume and AKD [56,57]. AKI accompanied by surgery may recover relatively quickly due to transient hemodynamic changes during surgery, while cardiac surgery is known to have a relationship with AKI [58]. Contrast-induced nephropathy often shows peak levels approximately 3-5 days after exposure and often returns to baseline within 7-14 days, indicating relatively good recovery [59,60]. Conversely, ANTIs are associated with intrinsic AKIs such as acute tubular necrosis and slower recovery despite expected renal function impairment [61-63]. A high urine specific gravity suggests dehydration, which can often be corrected through fluid supplementation alone, leading to fast recovery [64,65]. Indicators reflecting infection showed that higher values tended to be associated with good renal recovery, while the significant infection marker CRP yielded ambiguous results. Because the interpretation of our model aligns with that of previous clinical studies, the patterns learned by the model are reasonable. Although biomarkers such as cystatin-C are commonly suggested in various studies, they were not used in this study due to their low measurement frequency. Future research should expand the features used in this study to include such biomarkers [66-68].

### Limitations

This study had several limitations. First, owing to the lack of consensus on the AKI recovery criteria, the definitions had to rely on previous research findings. Second, there was a lack of data related to dialysis or kidney transplantation, which was addressed by excluding patients with an eGFR<60 mL/minute. As a result, caution is required when applying and interpreting the model for patients with preexisting chronic kidney disease or poor kidney function from the initial stages of hospitalization. However, the primary aim of this study was to predict “unexpected AKI.” Therefore, the analysis focused on patients with relatively preserved kidney function, who were considered to be at lower risk of AKI. Third, the study did not account for interventions or treatments before or post-AKI, which is crucial because patients’ preexisting conditions are significant factors in AKI and AKD. Fourth, in this study, the model was applied only in cases where AKI occurrences could be clearly identified, specifically when baseline SCr could be estimated at a specific time point and SCr measurements were available at that time. However, the proportion of AKI labels may differ from the actual occurrences. Therefore, despite the frequent measurement of SCr, caution is needed when applying and interpreting the model in situations where SCr measurements have not been conducted. Fifth, although our study used data extracted from different hospital information systems in various regions, it predominantly included data from Korean individuals. Therefore, we could not sufficiently consider racial diversity. Since there may be various differences, including kidney function, depending on ethnicity, future studies should include a multiethnic population.

### Conclusions

This study introduces a machine learning framework aimed at assisting in the early management of AKI in general ward patients. To develop the model, we used retrospective data from general wards, refining the operational definition of AKI and externally validating our approach. Our findings demonstrate that AI-driven methods can enhance risk stratification and enable timely interventions. Beyond improving predictive accuracy, this study underscores the potential of AI to streamline clinical workflows, optimize resource allocation, and ultimately reduce the burden of AKI-related complications. Integrating such models into routine hospital practice may support proactive decision-making, allowing physicians to implement tailored interventions based on individual patient risk profiles. Future research should focus on prospective validation, real-time clinical integration, and incorporating additional biomarkers to improve model generalizability and clinical relevance.

## Appendix

Multimedia Appendix 1 Labeling-related process.

Multimedia Appendix 2 Variable-related information.

Multimedia Appendix 3 Model Development and Validation Process.

Multimedia Appendix 4 Comparison before and after labeling improvement.

Multimedia Appendix 5 Acute Kidney Injury Early Prediction Model Results.

Multimedia Appendix 6 Acute Kidney Disease Early Prediction Model Results.

Multimedia Appendix 7 Framework to Assist in Managing Acute Kidney Injury.

## Abbreviations

AI: artificial intelligence
AKD: acute kidney disease
AKI: acute kidney injury
ANTI: nephrotoxic antibiotic
aPTT: activated partial thromboplastin time
AUROC: area under the receiver operating characteristic
BUN: blood urea nitrogen
CAT: categorical boosting
CECT: contrast-enhanced computed tomography
CRP: C-reactive protein
DBP: diastolic blood pressure
eGFR: estimated glomerular filtration rate
HR: hazard ratio
ICU: intensive care unit
KDIGO: Kidney Disease: Improving Global Outcomes
SCr: serum creatinine
SHAP: Shapley additive explanations
STROBE: Strengthening the Reporting of Observational Studies in Epidemiology
WBC: white blood cell

## Data Availability

The dataset used in this study and the models developed are not publicly available due to ethical restrictions, patient confidentiality, and institutional policies. However, they can be made available on reasonable request to the corresponding author.
