# Supplementary material for: Machine Learning to Assist in Managing Acute Kidney Injury in General Wards: Multicenter Retrospective Study
Source: J Med Internet Res. 2025 Mar 18;27:e66568. doi: 10.2196/66568 (PMC11962325; doi:10.2196/66568)
Supplement: Multimedia Appendix 1 [file jmir_v27i1e66568_app1.docx]

Figure S1. Comparison of the Previous KDIGO Criteria and the Improved Acute Kidney Injury Criteria Proposed in This Study


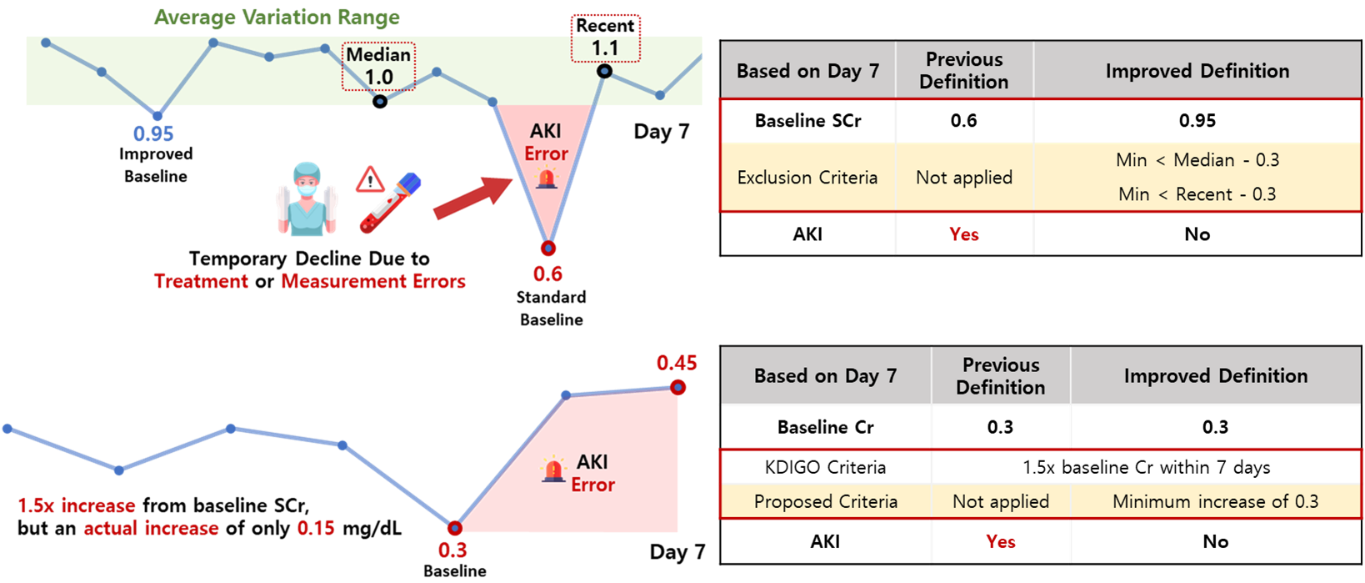


Comparison of the Previous KDIGO Criteria and the Improved AKI Criteria Proposed in This Study

1. **Previous KDIGO Criteria**

- Definition: AKI is defined as an increase in serum creatinine (SCr) by ≥0.3 mg/dL within 48 hours or an increase in SCr to ≥1.5 times the baseline within 7 days.
- Baseline SCr: Determined without specific considerations for transient fluctuations or measurement errors.
- Limitations: May classify patients with transient SCr fluctuations as AKI cases, leading to potential overestimation of AKI incidence.

1. **Improved AKI Criteria (Proposed in This Study)**

- Definition: Retains the KDIGO thresholds but introduces additional refinements to improve precision.
- Baseline SCr: Defined as the lowest SCr measured within the previous 7 days. If the baseline SCr is ≥0.3 mg/dL lower than the median or recent SCr values, it is excluded to avoid misclassification caused by temporary fluctuations or measurement errors.
- Minimum SCr Increase: Introduced a minimum increase threshold of 0.3 mg/dL to ensure appropriate identification and reduce false positives due to minor variations.
- Advantages: Reduces misclassification of transient cases, enhances specificity, and provides more accurate identification of high-risk patients.

Figure S2. Example of Acute Kidney Injury Labeling


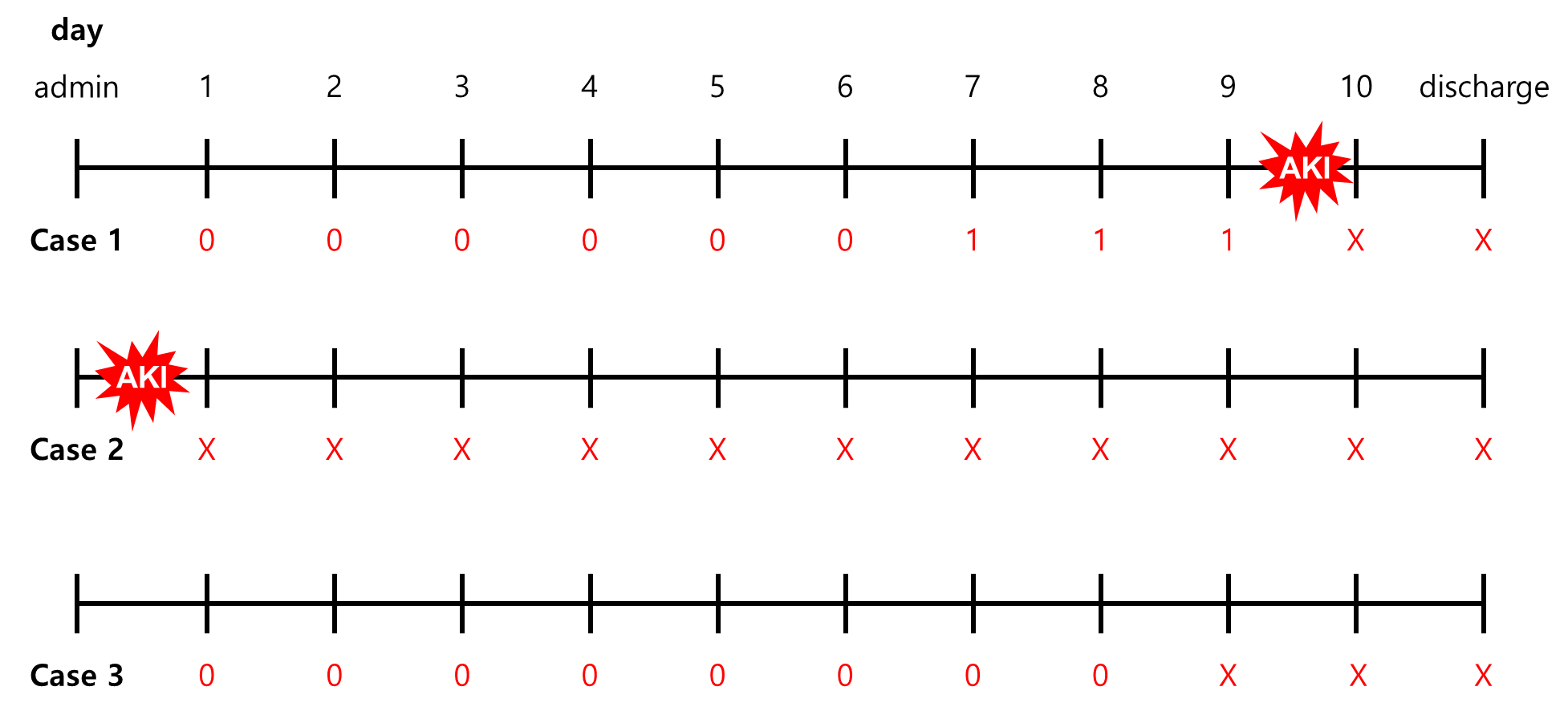


Case 1 refers to patients with acute kidney injury, Case 3 refers to patients without acute kidney injury. Case 2 includes patients, in whom acute kidney injury occurred on the first day and are excluded.

Figure S3. Example of Acute Kidney Disease Labeling


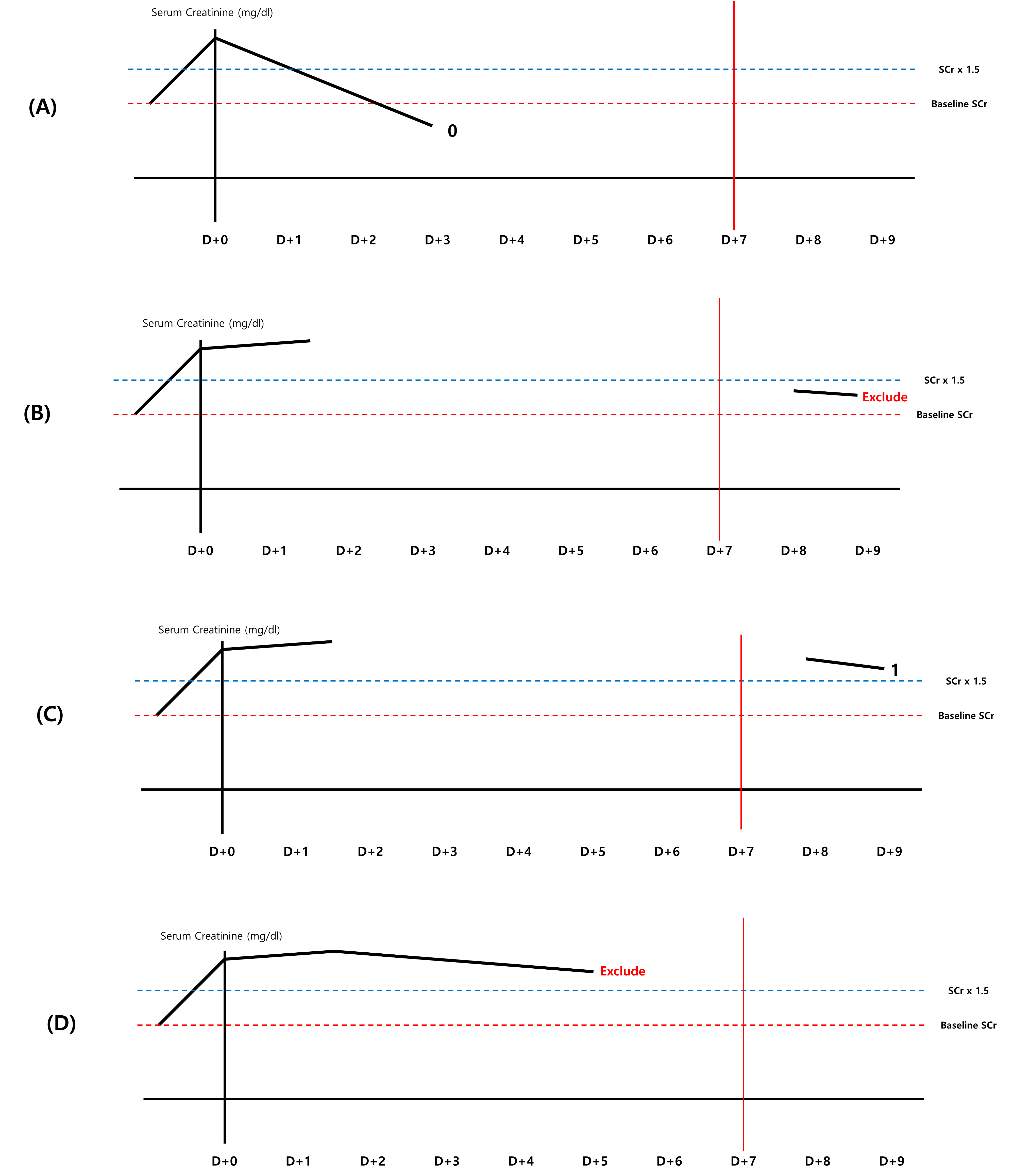


(A) and (B) show patients with and without acute kidney disease, respectively. (C) and (D) show examples in which acute kidney disease could not be clearly defined and are excluded. SCr, Serum Creatinine.
