# Supplementary material for: Machine Learning to Assist in Managing Acute Kidney Injury in General Wards: Multicenter Retrospective Study
Source: J Med Internet Res. 2025 Mar 18;27:e66568. doi: 10.2196/66568 (PMC11962325; doi:10.2196/66568)
Supplement: Multimedia Appendix 2 [file jmir_v27i1e66568_app2.docx]

Table S1. Employed Features

| **Category** | **Features** |
| --- | --- |
| Patient information  (2) | Sex, Age |
| Vital signs  (6) | Systolic Blood Pressure, Diastolic Blood Pressure, Heart Rate, Respiratory Rate, Body Temperature, Body Mass Index |
| Laboratory test  (27) | Hemoglobin, White Blood Cell Count, Platelet Count, Albumin, Glucose, Blood Urea Nitrogen, estimated Glomerular Filtration Rate, Total Bilirubin, Alanine Aminotransferase, Alkaline Phosphatase, Lactate Dehydrogenase, Uric acid, Triglycerides, Total Cholesterol, Phosphorus, Calcium, Sodium, Potassium, Chloride, Total Carbon Dioxide, C-Reactive Protein, pH, Partial Pressure of Carbon Dioxide, Partial Pressure of Oxygen, Urine Specific Gravity, Activated Partial Thromboplastin Time, N-Terminal pro-B-type Natriuretic Peptide |
| Other factors  (8) | Blood Sugar Test, BUN/Cr ratio, Nephrotoxic Antibiotics, Non-Steroidal Anti-Inflammatory Drugs, Cytotoxic Chemotherapeutic Agents, Contrast-Enhanced CT, General Anesthesia Surgery |

Table S2. Missing Data Counts Before and After Forward Filling

| **Features** | **Hospital A** | | **Hospital B** | | **Hospital C** | |
| --- | --- | --- | --- | --- | --- | --- |
|  | **Before** | **After** | **Before** | **After** | **Before** | **After** |
| BMI | 0.94 | 0.07 | 0.95 | 0.11 | 0.96 | 0.50 |
| Systolic BP | 0.04 | 0.00 | 0.03 | 0.00 | 0.01 | 0.00 |
| Diastolic BP | 0.04 | 0.01 | 0.03 | 0.01 | 0.01 | 0.00 |
| Heart rate | 0.02 | 0.01 | 0.03 | 0.01 | 0.01 | 0.00 |
| Respiratory rate | 0.02 | 0.01 | 0.03 | 0.01 | 0.01 | 0.00 |
| Body temperature | 0.02 | 0.01 | 0.02 | 0.00 | 0.00 | 0.00 |
| Hemoglobin | 0.52 | 0.00 | 0.52 | 0.00 | 0.52 | 0.00 |
| WBC count | 0.52 | 0.00 | 0.52 | 0.00 | 0.52 | 0.00 |
| Platelet | 0.52 | 0.00 | 0.52 | 0.00 | 0.52 | 0.00 |
| Albumin | 0.67 | 0.03 | 0.63 | 0.00 | 0.57 | 0.00 |
| Glucose | 0.89 | 0.04 | 0.84 | 0.03 | 0.57 | 0.07 |
| BUN | 0.60 | 0.00 | 0.61 | 0.00 | 0.56 | 0.03 |
| Serum Creatinine | 0.59 | 0.00 | 0.61 | 0.00 | 0.56 | 0.00 |
| Total bilirubin | 0.66 | 0.01 | 0.63 | 0.00 | 0.57 | 0.00 |
| ALT | 0.62 | 0.00 | 0.62 | 0.00 | 0.57 | 0.00 |
| ALP | 0.79 | 0.11 | 0.65 | 0.01 | 0.57 | 0.43 |
| LDH | 0.95 | 0.47 | 0.92 | 0.45 | 0.88 | 0.00 |
| Uric acid | 0.93 | 0.37 | 0.89 | 0.42 | 0.86 | 0.00 |
| Triglycerides | 0.98 | 0.59 | 0.98 | 0.63 | 0.88 | 0.23 |
| Total cholesterol | 0.88 | 0.29 | 0.69 | 0.03 | 0.88 | 0.00 |
| Phosphorus | 0.85 | 0.29 | 0.81 | 0.26 | 0.83 | 0.00 |
| Calcium | 0.81 | 0.11 | 0.76 | 0.04 | 0.84 | 0.07 |
| Sodium | 0.57 | 0.00 | 0.56 | 0.00 | 0.55 | 0.21 |
| Potassium | 0.57 | 0.00 | 0.56 | 0.00 | 0.55 | 0.05 |
| Chloride | 0.57 | 0.00 | 0.57 | 0.00 | 0.55 | 0.00 |
| Total CO_2_ | 0.75 | 0.21 | 0.87 | 0.37 | 0.69 | 0.05 |
| C-reactive protein | 0.90 | 0.32 | 0.68 | 0.01 | 0.69 | 0.04 |
| pH | 0.89 | 0.41 | 0.96 | 0.87 | 0.88 | 0.00 |
| pCO2 | 0.89 | 0.41 | 0.96 | 0.87 | 0.88 | 0.05 |
| pO2 | 0.76 | 0.02 | 0.76 | 0.02 | 0.87 | 0.43 |
| Urine SG | 0.81 | 0.06 | 0.85 | 0.06 | 0.88 | 0.43 |
| aPTT | 0.98 | 0.62 | 0.97 | 0.50 | 0.95 | 0.43 |
| Pro-BNP | 0.66 | 0.34 | 0.63 | 0.37 | 0.65 | 0.03 |
| BST | 0.94 | 0.07 | 0.95 | 0.11 | 0.96 | 0.44 |

Hospital A, Korea University Anam Hospital; Hospital B, Korea University Guro Hospital; and Hospital C, Soonchunhyang University Cheonan Hospital; BP, blood pressure; WBC, white blood cell; BUN, blood urea nitrogen; eGFR, estimated glomerular filtration rate; ALT, alanine aminotransferase; ALP, alkaline phosphatase; LDH, lactate dehydrogenase; SG, specific gravity; BNP, brain natriuretic peptide; BST, blood sugar test; pCO_2_, partial pressure of carbon dioxide; pO_2_, partial pressure of oxygen; aPTT, activated partial thromboplastin time

Table S3. Application of Missing Indicator Method

| **Features** | **Category 1** | **Category 2** | **Category 3** | **Category 4** |
| --- | --- | --- | --- | --- |
| BST (mg/dL) | ≤200 | >200 | Missing |  |
| LDH (U/L) | ≤500 | >500 | Missing |  |
| Uric acid (mg/dL) | ≤7 | >7 | Missing |  |
| Triglycerides (mg/dL) | <100 | 100–200 | >200 | Missing |
| Total cholesterol (mg/dL) | <100 | 100–200 | >200 | Missing |
| Phosphorus (mg/dL) | <2.5 | 2.5–5.5 | >5.5 | Missing |
| C-reactive protein (mg/L) | <10 | 10–50 | >50 | Missing |
| pH | <7.35 | 7.35–7.45 | >7.45 | Missing |
| Total CO2 (mmol/L) | <20 | 20–25 | >25 | Missing |
| pCO2 (mmHg) | ≤40 | Missing | >40 |  |
| pO2 (mmHg) | ≤80 | Missing | >80 |  |
| Pro-BNP (pg/mL) | <250 | 250–1,000 | >1,000 | Missing |

BST, blood sugar test; LDH, lactate dehydrogenase; BNP, brain natriuretic peptide; pCO2, partial pressure of carbon dioxide; pO2, partial pressure of oxygen
