# Supplementary material for: Machine Learning to Assist in Managing Acute Kidney Injury in General Wards: Multicenter Retrospective Study
Source: J Med Internet Res. 2025 Mar 18;27:e66568. doi: 10.2196/66568 (PMC11962325; doi:10.2196/66568)
Supplement: Multimedia Appendix 3 [file jmir_v27i1e66568_app3.docx]

Figure S4. Model Development and Validation Process


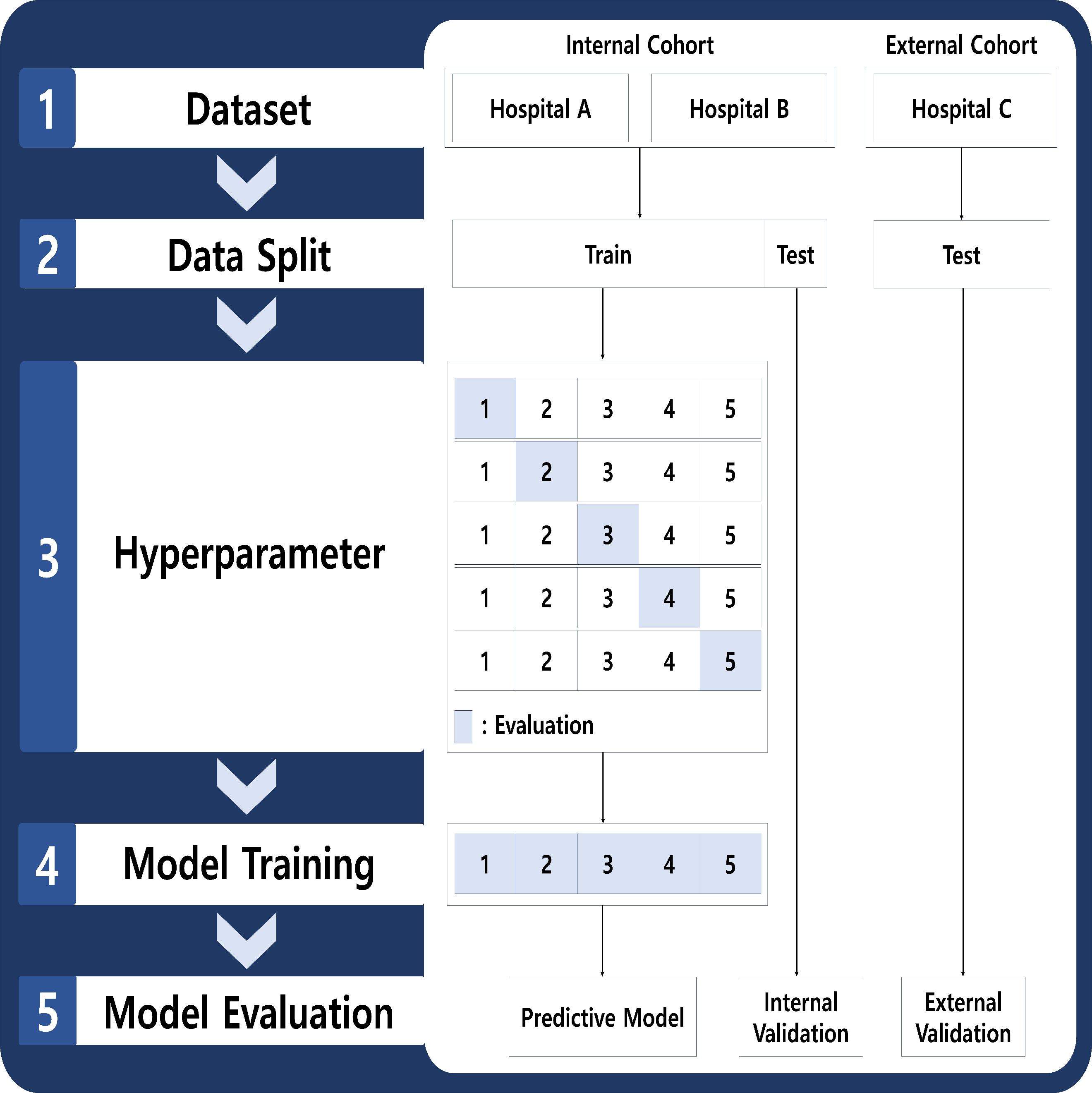


Hospital A: Korea University Anam Hospital; Hospital B: Korea University Guro Hospital; and Hospital C: Soonchunhyang University Cheonan Hospital, each utilizing different hospital information systems. External validation is conducted at Hospital C due to regional differences. Data from two hospitals at the Korea University are used for training and internal validation. To prevent data leakage, the patients are randomly divided into six groups based on event rates to ensure that the data from the same patient do not overlap between training and evaluation. Groups 1–5 are used for hyperparameter tuning and training. All pre-processing during training, including undersampling, is conducted solely on the training data. Model evaluation presents the mean and standard deviation of the cross-validation results across the five groups, followed by training on all five groups, internal validation on Group 6 of Korea University, and external validation of all data from Hospital C.

The dataset was randomly divided into six groups by patient, ensuring the same label ratio across groups. The final group (Group 6) was used for internal validation, whereas the remaining five groups were used for cross-validation in hyperparameter tuning and model selection. Over five iterations, four groups were used for training and one group was used for early stopping and performance comparison. A grid search was performed to tune the hyperparameters. The processes of early stopping, hyperparameter tuning, and model selection were based on area under the receiver operating characteristic curve. For the early AKI prediction model, random undersampling was applied to the training data at a 10:1 ratio.
