# Supplementary material for: Machine Learning to Assist in Managing Acute Kidney Injury in General Wards: Multicenter Retrospective Study
Source: J Med Internet Res. 2025 Mar 18;27:e66568. doi: 10.2196/66568 (PMC11962325; doi:10.2196/66568)
Supplement: Multimedia Appendix 4 [file jmir_v27i1e66568_app4.docx]

Note S1. Comparison of Hospital Stay Duration, Incidence Rates, and Outcomes Between the Early Acute Kidney Injury Prediction Model and Actual Labeling

Calibration plots were used to assess the agreement between predicted probabilities and observed outcomes. These plots compare the predicted risk of acute kidney injury (AKI) or acute kidney disease (AKD) against the actual observed incidence across different risk strata. A perfectly calibrated model would show a plot where the predicted probabilities align closely with the 45-degree line. Calibration was evaluated for both internal and external validation cohorts to ensure that the model maintained its predictive performance across different patient populations and settings. Calibration plots were generated for both internal and external cohorts to evaluate the models.

The Cox proportional hazards model was used to compare hazard ratios between patients with actual AKD and those predicted by the model. The Cox proportional hazards model is widely used in survival analysis to examine the association between the survival time of patients and one or more predictor variables. The Cox model is appropriate for this analysis as it allows for the inclusion of both time-dependent covariates and censored data, providing a robust assessment of the model’s predictive performance over time. The proportional hazards assumption was tested to ensure the validity of the model, and the results were interpreted using 95% confidence intervals. Cox proportional hazards analysis was performed to obtain adjusted hazard ratios comparing actual patients with AKD and those predicted to develop AKD by the model against patients not predicted to develop AKD. To compare the results, this analysis was performed without the improved AKI labeling.

The previous criteria applied the Kidney Disease: Improving Global Outcomes guidelines uniformly and set the baseline serum creatinine (SCr) using the minimum value. If no baseline SCr levels were available, the most recent value measured within 180 days was used. Additionally, the minimum increase of 0.3 or more required to identify AKI by the 1.5 times criterion is not applied, and a sudden decrease in SCr also affects the determination of the baseline SCr. There was no significant difference in the duration of hospital stay between the two groups. However, in terms of the incidence rates, the developed model identified more patients with AKI than the actual incidence. Hazard Ratios were calculated using the Cox proportional hazards analysis of patients with AKI in each cohort, focusing on those who experienced AKI and were hospitalized for > 7, 30, or 90 days. An event was defined as a 30% (or 40%) or greater decrease in eGFR within each day compared with the eGFR at the time of AKI occurrence.

Table S4. Patient Counts and Basic Statistics Based on Labeling Criteria

| **Features** | **Acute Kidney Injury** | | | | |  |
| --- | --- | --- | --- | --- | --- | --- |
|  | **Previous (*n* = 19,180) ^a)^** | | **Current (*n* = 10,556) ^b)^** | | ***P*-value** |  |
| Incident Rate, % | 14.20 | | 7.82 | | <.001^d)^ |  |
| Age, year | 64.16±15.35 | | 65.29±14.83 | | <.001^d)^ |  |
| Male, % | 56.88 | | 66.29 | | <.001^d)^ |  |
| Serum Cr, mg/dL | 0.90 (0.60, 1.20) | | 1.19 (0.99, 1.50) | | <.001^d)^ |  |
| eGFR, mL/min | 82.22±28.96 | | 61.80±22.34 | | <.001^d)^ |  |
| Serum Cr change, mg/dL ^c)^ | 0.23 (0.10, 0.42) | | 0.42 (0.28, 0.68) | | <.001^d)^ |  |
| **Features** | | **Acute Kidney Disease** | | | | |
|  |  | **Previous (*n* = 1,432) ^a)^** | | **Current (*n* = 1,278) ^b)^** | | ***P*-value** |
| Incident Rate | | 9.41 | | 16.66 | | <.001^d)^ |
| Age, year | | 64.13±14.40 | | 64.28±14.46 | | <.001^d)^ |
| Male, % | | 67.53 | | 67.84 | | .89 |
| Serum Cr, mg/dL | | 1.10 (0.84, 1.52) | | 1.23 (1.00, 1.74) | | <.001^d)^ |
| eGFR, mL/min | | 68.05±28.84 | | 58.59±24.13 | | <.001^d)^ |
| Serum Cr change, mg/dL ^c)^ | | 0.40 (0.21, 0.74) | | 0.54 (0.35, 0.96) | | <.001^d)^ |

^a)^ Baseline Cr is imputed with the most recent value between 7-180 days if missing, without attempting any improvements based on the KDIGO criteria. ^b)^ In contrast, using the method applied in this paper, we aim to prevent identification of acute kidney injury due to simple fluctuations in patients with low serum creatinine levels. Both acute kidney injury and acute kidney disease represent statistics based on data from the day of acute kidney injury. ^c)^ Difference between the median of previous values and serum creatinine levels on the day of acute kidney injury. ^d)^ indicate statistically significant.

Table S5. Evaluation Results of the Acute Kidney Injury Model Developed Based on Previous Labeling Criteria

| **Validation** | **Model** | **Accuracy** | **Precision** | **Recall** | **F1** | **AUROC** | **AUPRC** |
| --- | --- | --- | --- | --- | --- | --- | --- |
| Cross-  validation | LR | 0.9245  (0.0008) | 0.5568  (0.0194) | 0.0478  (0.0034) | 0.0879  (0.0058) | 0.7230  (0.0086) | 0.2251  (0.0040) |
|  | RF | 0.9628  (0.0007) | 0.9943  (0.0025) | 0.5151  (0.0101) | 0.6786  (0.0088) | 0.9003  (0.0018) | 0.7274  (0.0065) |
|  | XGB | 0.9627  (0.0009) | 0.9148  (0.0054) | 0.5641  (0.0112) | 0.6978  (0.0089) | 0.8997  (0.0042) | 0.7250  (0.0052) |
|  | LGBM | 0.9640  (0.0009) | 0.9551  (0.0027) | 0.5535  (0.0123) | 0.7008  (0.0102) | 0.9056  (0.0027) | 0.7341  (0.0052) |
|  | CAT | 0.9643  (0.0009) | 0.9839  (0.0013) | 0.5405  (0.0118) | 0.6976  (0.0099) | 0.9057  (0.0026) | 0.7348  (0.0045) |
| Internal | LR | 0.9227 | 0.4973 | 0.0383 | 0.0711 | 0.7161 | 0.2101 |
|  | RF | 0.9621 | 0.9959 | 0.5114 | 0.6758 | 0.8990 | 0.7226 |
|  | XGB | 0.9617 | 0.9107 | 0.5586 | 0.6924 | 0.9050 | 0.7261 |
|  | LGBM | 0.9630 | 0.9549 | 0.5465 | 0.6952 | 0.9090 | 0.7318 |
|  | CAT | 0.9634 | 0.9818 | 0.5357 | 0.6932 | 0.9079 | 0.7307 |
| External | LR | 0.9319 | 0.4496 | 0.0199 | 0.0382 | 0.6926 | 0.1642 |
|  | RF | 0.9639 | 0.9968 | 0.4682 | 0.6371 | 0.8656 | 0.6549 |
|  | XGB | 0.9614 | 0.8819 | 0.4969 | 0.6357 | 0.8650 | 0.6504 |
|  | LGBM | 0.9626 | 0.9245 | 0.4873 | 0.6382 | 0.8704 | 0.6535 |
|  | CAT | 0.9635 | 0.9705 | 0.4760 | 0.6387 | 0.8692 | 0.6534 |

LR, Logistic Regression; RF, Random Forest; XGB, eXtreme Gradient Boosting; LGBM, Light Gradient Boosting Machine; CAT, Categorical Boosting; AUROC, area under the receiver operating characteristic curve; AUPRC, area under the precision-recall curve.

Table S6. Evaluation Results of the Acute Kidney Disease Model Developed Based on Previous Labeling Criteria

| **Validation** | **Model** | **Accuracy** | **Precision** | **Recall** | **F1** | **AUROC** | **AUPRC** |
| --- | --- | --- | --- | --- | --- | --- | --- |
| Cross-  validation | LR | 0.9080  (0.0068) | 0.5888  (0.1011) | 0.1322  (0.0192) | 0.2146  (0.0277) | 0.7787  (0.0169) | 0.3497  (0.0287) |
|  | RF | 0.9062  (0.0058) | 0.7324  (0.1498) | 0.0288  (0.0093) | 0.0549  (0.0171) | 0.7727  (0.0198) | 0.3301  (0.0318) |
|  | XGB | 0.9073  (0.0055) | 0.5547  (0.0388) | 0.1546  (0.0214) | 0.2405  (0.0197) | 0.7632  (0.0197) | 0.3386  (0.0350) |
|  | LGBM | 0.9086  (0.0045) | 0.5790  (0.0636) | 0.1412  (0.0194) | 0.2270  (0.0296) | 0.7645  (0.0159) | 0.3425  (0.0274) |
|  | CAT | 0.9078  (0.0054) | 0.7024  (0.0970) | 0.0536  (0.0143) | 0.0995  (0.0252) | 0.0995  (0.0135) | 0.3670  (0.0345) |
| Internal | LR | 0.9018 | 0.6000 | 0.0802 | 0.1415 | 0.7680 | 0.3125 |
|  | RF | 0.9018 | 1.0000 | 0.0267 | 0.0521 | 0.7826 | 0.3442 |
|  | XGB | 0.9007 | 0.5366 | 0.1176 | 0.1930 | 0.7490 | 0.3395 |
|  | LGBM | 0.9066 | 0.7188 | 0.1230 | 0.2100 | 0.7707 | 0.3425 |
|  | CAT | 0.9034 | 0.8333 | 0.0535 | 0.1005 | 0.7857 | 0.3595 |
| External | LR | 0.9112 | 0.5000 | 0.0610 | 0.1087 | 0.7689 | 0.3050 |
|  | RF | 0.9112 | 0.5000 | 0.0159 | 0.0308 | 0.7825 | 0.3198 |
|  | XGB | 0.9124 | 0.5373 | 0.0955 | 0.1622 | 0.7649 | 0.3106 |
|  | LGBM | 0.9114 | 0.5070 | 0.0955 | 0.1607 | 0.7805 | 0.3189 |
|  | CAT | 0.9131 | 0.6667 | 0.0424 | 0.0798 | 0.7893 | 0.3445 |

LR, Logistic Regression; RF, Random Forest; XGB, eXtreme Gradient Boosting; LGBM, Light Gradient Boosting Machine; CAT, Categorical Boosting; AUROC, area under the receiver operating characteristic curve; AUPRC, area under the precision-recall curve.

Table S7. Comparison of Models Based on Labeling Criteria

| **Valid Label** | | **Train Label** | | **Accuracy** | | **Precision** | | **Recall** | **F1** | **AUROC** | | **AUPRC** |
| --- | --- | --- | --- | --- | --- | --- | --- | --- | --- | --- | --- | --- |
| Acute  Kidney  Injury | Previous | Previous | | 0.9220 | | 0.4473 | | 0.6228 | 0.5207 | 0.8692 | | 0.6534 |
|  |  | Current | | 0.9044 | | 0.3703 | | 0.5869 | 0.4541 | 0.8021 | | 0.4464 |
|  | Current | Previous | | 0.9210 | | 0.2875 | | 0.6173 | 0.3923 | 0.8534 | | 0.4068 |
|  |  | Current | | 0.9217 | | 0.3004 | | 0.6742 | 0.4156 | 0.8860 | | 0.6290 |
|  | Only Previous | Previous | | 0.9370 | | 0.2885 | | 0.6172 | 0.3932 | 0.8747 | | 0.6027 |
|  |  | Current | | 0.9193 | | 0.2038 | | 0.4957 | 0.2889 | 0.7191 | | 0.1755 |
| Acute  Kidney  Disease | Previous | Previous | | 0.7991 | | 0.2479 | | 0.6207 | 0.3543 | 0.7893 | | 0.3445 |
|  |  | Current | | 0.8349 | | 0.2686 | | 0.4987 | 0.3491 | 0.7663 | | 0.3184 |
|  | Current | Previous | | 0.7508 | | 0.3339 | | 0.5864 | 0.4255 | 0.7492 | | 0.4125 |
|  |  | Current | | 0.7946 | | 0.3958 | | 0.5802 | 0.4706 | 0.7833 | | 0.4368 |
| Only Previous | **Age,**  **year** | | **Male,**  **%** | | **Serum Cr,**  **mg/dL** | | **eGFR,**  **mL/min** | | | | **Serum Cr change, mg/dL** | |
|  | 62.51 ± 15.99 | | 40.85 | | 0.6 (0.51, 0.80) | | 100.35 (18.94) | | | | 0.10 (0.00, 0.20) | |

AUROC, area under the receiver operating characteristic curve; AUPRC, area under the precision-recall curve

External validation data identified 5,407 patients with AKI according to the previous criteria, whereas 2,898 patients were identified using the newly defined criteria. Consequently, 2,509 patients were classified as having AKI according to the previous criteria, but not under the new criteria. A model trained on data labeled according to the previous criteria showed a similar performance for these patients, whereas a model trained on the new criteria did not.

Under the previous criteria, of the 11,181 alarms triggered, 4,961 (44.37%) were for patients with ambiguous AKI. A model trained on the previous criteria correctly alarmed 6,963 out of 9,514 early alarms triggered for patients identified as having AKI, with 73.19% accuracy. Among patients identified as having AKI only under the previous criteria, the model correctly alarmed 3,062 out of 4,587 alarms triggered with an accuracy of 66.75 %.

Table S8. Comparison of Hazard Ratios for Renal Function Decline Based on the Occurrence of Acute Kidney Disease

| **Criteria Duration, days** | **Criteria eGFR, mL/min** | **Variables** | **Hazard Ratios (95% CI)** | |
| --- | --- | --- | --- | --- |
|  |  |  | **Unadjusted ^a)^** | **Adjusted ^b)^** |
| 7 | $\geq$30 % decrease | Current AKI Prediction ^a)^ | 3.09 (2.44-3.92) | 3.03 (2.39-3.85) |
|  |  | Previous AKI Prediction ^b)^ | 2.31 (1.82-2.92) | 2.32 (1.83-2.94) |
|  |  | Current AKI Criteria ^c)^ | 10.23 (7.96-13.14) | 10.34 (8.02-13.34) |
|  |  | Previous AKI Criteria ^d)^ | 8.71 (6.87-11.06) | 8.62 (6.77-10.97) |
|  | $\geq$40 % decrease | Current AKI Prediction ^a)^ | 3.28 (2.50-4.31) | 3.20 (2.43-4.20) |
|  |  | Previous AKI Prediction ^b)^ | 2.70 (2.04-3.58) | 2.71 (2.05-3.60) |
|  |  | Current AKI Criteria ^c)^ | 11.42 (8.51-15.32) | 11.47 (8.51-15.46) |
|  |  | Previous AKI Criteria ^d)^ | 9.82 (7.40-13.03) | 9.68 (7.27-12.89) |
| 30 | $\geq$30 % decrease | Current AKI Prediction ^a)^ | 1.98 (1.35-2.91) | 2.03 (1.38-3.00) |
|  |  | Previous AKI Prediction ^b)^ | 1.63 (1.15-2.30) | 1.66 (1.17-2.34) |
|  |  | Current AKI Criteria ^c)^ | 5.12 (3.48-7.53) | 4.98 (3.35-7.40) |
|  |  | Previous AKI Criteria ^d)^ | 4.07 (2.84-5.83) | 3.88 (2.70-5.60) |
|  | $\geq$40 % decrease | Current AKI Prediction ^a)^ | 2.02 (1.31-3.12) | 2.05 (1.32-3.17) |
|  |  | Previous AKI Prediction ^b)^ | 1.64 (1.10-2.44) | 1.70 (1.14-2.54) |
|  |  | Current AKI Criteria ^c)^ | 4.88 (3.16-7.53) | 4.61 (2.96-7.18) |
|  |  | Previous AKI Criteria ^d)^ | 3.77 (2.50-5.68) | 3.49 (2.30-5.28) |
| 90 | $\geq$30 % decrease | Current AKI Prediction ^a)^ | 1.48 (0.68-3.22) | 1.54 (0.70-3.41) |
|  |  | Previous AKI Prediction ^b)^ | 1.26 (0.69-.2.32) | 1.21 (0.65-2.23) |
|  |  | Current AKI Criteria ^c)^ | 3.71 (1.61-8.58) | 3.40 (1.43-8.09) |
|  |  | Previous AKI Criteria ^d)^ | 2.08 (0.96-4.48) | 1.85 (0.84-4.11) |
|  | $\geq$40 % decrease | Current AKI Prediction ^a)^ | 1.39 (0.60-3.26) | 1.46 (0.61-3.49) |
|  |  | Previous AKI Prediction ^b)^ | 1.41 (0.72-2.78) | 1.42 (0.71-2.84) |
|  |  | Current AKI Criteria ^c)^ | 1.86 (0.69-5.05) | 1.70 (0.59-4.84) |
|  |  | Previous AKI Criteria ^d)^ | 1.24 (0.48-3.21) | 1.13 (0.43-2.99) |

AKI, acute kidney injury; AKD, acute kidney disease; a) results derived from the model developed with refined criteria; and c) outcomes derived from actual patients with AKI and AKD. Both b) and d) apply the previous criteria, utilizing the last measurement within 180 days for cases with missing data, thereby altering the operational definition of labeling, and performing the same process. a) presents unadjusted Hazard Ratios, considering only the presence of AKD. b) presents adjusted Hazard Ratios, accounting for gender, age, eGFR levels, and changes in creatinine.

In most cases, it was observed that the hazard ratio for renal function decline, based on the current criteria for identifying patients with acute kidney injury, is higher when considering the presence of acute kidney disease.

Figure S5. Serum Creatinine Trends for Patients Meeting Only the Previous Acute Kidney Injury Criteria


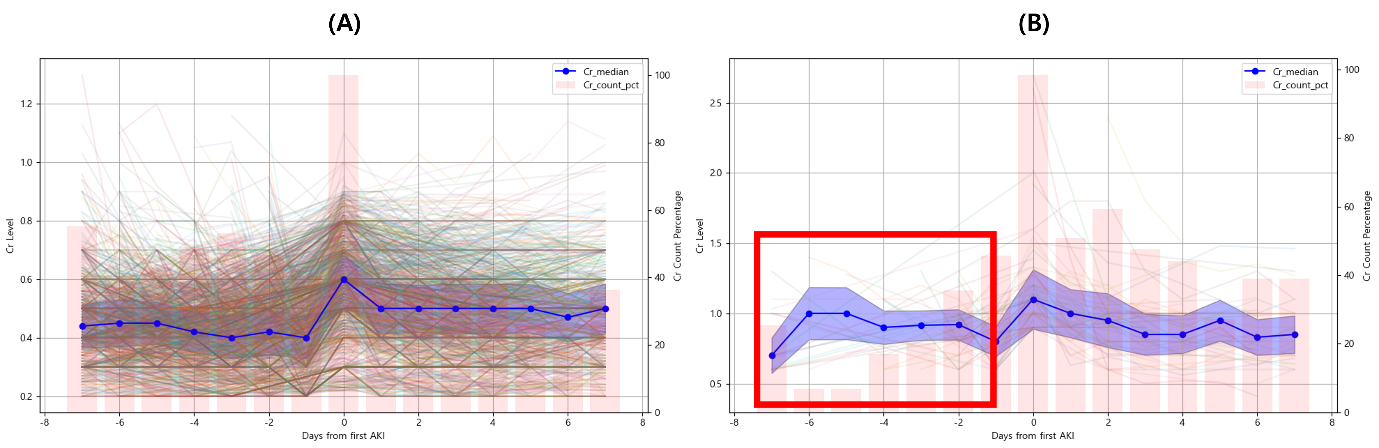


(A) Results for 2,242 patients with a baseline serum creatinine $<$ 0.6 mg/dL. (B) Results for 267 patients with a baseline serum creatinine $\geq$ 0.6 mg/dL. As seen in the red box, patients in (B) mostly experienced a decrease in serum creatinine prior to the onset of AKI. AKI, acute kidney injury
