# Supplementary material for: Machine Learning to Assist in Managing Acute Kidney Injury in General Wards: Multicenter Retrospective Study
Source: J Med Internet Res. 2025 Mar 18;27:e66568. doi: 10.2196/66568 (PMC11962325; doi:10.2196/66568)
Supplement: Multimedia Appendix 5 [file jmir_v27i1e66568_app5.docx]

Figure S6. Distribution of Acute Kidney Injury by Cohort


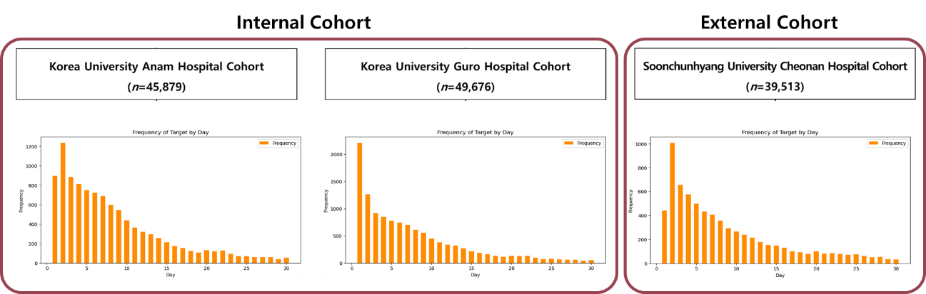


Table S9. Basic Statistics for Acute Kidney Injury in the Internal Cohort

| **Features** | **Non-AKI (*n* = 87,897)** | **AKI (*n* = 7,658)** | ***P*-value** |
| --- | --- | --- | --- |
| Age, year | 61.41±16.27 | 65.15±14.65 | <.001^a)^ |
| Male | 49,596 (56.43) | 5,083 (66.38) | <.001^a)^ |
| Systolic BP, mmHg | 115.90±26.96 | 105.01±38.58 | <.001^a)^ |
| Diastolic BP, mmHg | 72.98±10.78 | 69.53±14.41 | <.001^a)^ |
| Heart rate, bpm | 77.32±13.00 | 93.31±21.83 | <.001^a)^ |
| Respiratory rate, bpm | 20.0 (18.0,20.0) | 20.0 (18.0,20.0) | <.001^a)^ |
| Body temperature, °C | 36.74±0.37 | 36.94±0.69 | <.001^a)^ |
| Hemoglobin, g/dL | 11.39±1.92 | 10.32±2.24 | <.001^a)^ |
| WBC count, 10³/μL | 7.32±6.67 | 13.58±13.65 | <.001^a)^ |
| Platelet, 10³/μL | 250.41±120.82 | 182.11±124.70 | <.001^a)^ |
| Albumin, g/dL | 3.49±0.50 | 3.00±0.61 | <.001^a)^ |
| BUN, mg/dL | 12.9 (9.7,17.0) | 30.0 (21.1,42.5) | <.001^a)^ |
| Serum Cr, mg/dL | 0.7 (0.5,0.8) | 1.2 (1.0,1.5) | <.001^a)^ |
| BUN/Cr ratio | 22.52±14.13 | 27.15±14.60 | <.001^a)^ |
| eGFR, mL/min | 102.38±16.64 | 61.50±22.12 | <.001^a)^ |
| Glucose, mg/dL | 116.0 (99.0,145.0) | 136.5 (109.0,183.0) | <.001^a)^ |
| Total bilirubin, mg/dL | 0.6 (0.4,0.8) | 0.9 (0.6,1.8) | <.001^a)^ |
| ALP, IU/L | 81.0 (62.0,112.0) | 94.0 (66.0,155.0) | <.001^a)^ |
| ALT, IU/L | 21.0 (14.0,37.0) | 27.0 (15.0,55.5) | <.001^a)^ |
| BST, mg/dL | 131.0 (109.0,167.0) | 150.0 (120.0,197.0) | <.001^a)^ |
| Uric acid, mg/dL | 4.0 (2.9,5.3) | 4.6 (3.2,6.3) | <.001^a)^ |
| Triglycerides, mg/dL | 101.0 (71.0,148.0) | 100.0 (70.0,151.0) | .85 |
| Total cholesterol, mg/dL | 144.0 (117.0,175.0) | 122.0 (92.0,158.0) | <.001^a)^ |
| Calcium, mg/dL | 8.62±0.66 | 8.26±1.01 | <.001^a)^ |

Table S9 (continuation). Basic Statistics for Acute Kidney Injury in the Internal Cohort

| **Features** | **Non-AKI (*n* = 87,897)** | **AKI (*n* = 7,658)** | ***P*-value** |
| --- | --- | --- | --- |
| Phosphorus, mg/dL | 3.4 (2.9,3.8) | 3.6 (2.9,4.5) | <.001^a)^ |
| Sodium, mmol/L | 138.13±3.58 | 138.16±7.99 | .49 |
| Potassium, mmol/L | 4.06±0.46 | 4.16±0.81 | <.001^a)^ |
| Chloride, mmol/L | 103.57±4.03 | 103.94±8.05 | <.001^a)^ |
| Urine SG | 1.02±0.01 | 1.02±0.01 | <.001^a)^ |
| aPTT, sec | 37.47±8.57 | 43.40±17.80 | <.001^a)^ |
| LDH, U/L | 417.0 (339.0,551.0) | 552.0 (412.0,868.0) | <.001^a)^ |
| C-reactive protein, mg/L | 11.0 (2.6,39.1) | 60.5 (15.7,145.4) | <.001^a)^ |
| pH | 7.4 (7.4,7.5) | 7.4 (7.4,7.5) | <.001^a)^ |
| pCO2, mmHg | 34.2 (30.0,38.7) | 34.0 (28.9,40.0) | .08 |
| pO2, mmHg | 92.5 (78.4,116.0) | 91.9 (76.0,123.5) | .21 |
| Total CO2, mmol/L | 25.0 (23.0,27.1) | 22.3 (19.0,25.9) | <.001^a)^ |
| Pro-BNP, pg/mL | 245.9 (82.8,799.6) | 695.0 (199.9,2325.0) | <.001^a)^ |
| Nephrotoxic antibiotics† | 10,301 (11.72) | 2,095 (27.36) | <.001^a)^ |
| NSAIDs† | 31,178 (35.47) | 2,642 (34.5) | .09 |
| CHEMOs† | 9,415 (10.71) | 767 (10.02) | .06 |
| Contrast-enhanced CT† | 27,098 (30.83) | 3,365 (43.94) | <.001^a)^ |
| general anesthesia† | 13,504 (15.36) | 2,070 (27.03) | <.001^a)^ |

Data are presented as mean ± standard deviation, median (interquartile range), or count (%) as appropriate. These features are checked for exposure within one week. ^a)^*P* <0.05. AKI, acute kidney injury; BP, blood pressure; WBC, white blood cell; BUN, blood urea nitrogen; Cr, creatinine; eGFR, estimated glomerular filtration rate; ALT, alanine aminotransferase; ALP, alkaline phosphatase; aPTT, activated partial thromboplastin time; LDH, lactate dehydrogenase; SG, specific gravity; BNP, brain natriuretic peptide; BST, blood sugar test; NSAIDs, non-steroidal anti-inflammatory drugs; CHEMOs, cytotoxic chemotherapeutic agents, CT, computed tomography; pCO2, partial pressure of carbon dioxide; pO2, partial pressure of oxygen. ^a)^ indicate statistically significant.

Table S10. Basic Statistics for Acute Kidney Injury in the External Cohort

| **Features** | **Non-AKI (*n* = 36,615)** | **AKI (*n* = 2,898)** | ***P*-value** |
| --- | --- | --- | --- |
| Age, year | 61.15±16.64 | 65.66±15.30 | <.001^a)^ |
| Male | 20,342 (55.56) | 1,915 (66.08) | <.001^a)^ |
| Systolic BP, mmHg | 118.10±14.02 | 128.91±22.51 | <.001^a)^ |
| Diastolic BP, mmHg | 72.75±9.71 | 78.92±12.85 | <.001^a)^ |
| Heart rate, bpm | 77.31±12.33 | 104.34±26.81 | <.001^a)^ |
| Respiratory rate, bpm | 18.0 (16.0,18.0) | 20.0 (18.0,26.0) | <.001^a)^ |
| Body temperature, °C | 36.69±0.38 | 37.33±0.78 | <.001^a)^ |
| Hemoglobin, g/dL | 11.56±1.83 | 10.79±2.25 | <.001^a)^ |
| WBC count, 10³/μL | 7.12±4.11 | 12.83±12.03 | <.001^a)^ |
| Platelet, 10³/μL | 248.78±111.54 | 202.22±120.75 | <.001^a)^ |
| Albumin, g/dL | 3.54±0.55 | 2.96±0.72 | <.001^a)^ |
| BUN, mg/dL | 11.2 (8.3,14.9) | 24.0 (16.4,36.1) | <.001^a)^ |
| Serum Cr, mg/dL | 0.7 (0.5,0.8) | 1.2 (1.0,1.5) | <.001^a)^ |
| BUN/Cr ratio | 18.92±10.35 | 22.56±12.65 | <.001^a)^ |
| eGFR, mL/min | 102.49±15.96 | 62.62±22.90 | <.001^a)^ |
| Glucose, mg/dL | 99.0 (89.0,117.0) | 129.0 (103.0,173.0) | <.001^a)^ |
| Total bilirubin, mg/dL | 0.4 (0.3,0.6) | 0.7 (0.4,1.3) | <.001^a)^ |
| ALP, IU/L | 73.0 (57.0,100.0) | 85.0 (61.0,139.0) | <.001^a)^ |
| ALT, IU/L | 19.0 (13.0,33.0) | 24.0 (14.0,49.0) | <.001^a)^ |
| BST, mg/dL | 138.3 (116.8,171.5) | 151.5 (124.2,198.0) | <.001^a)^ |
| Uric acid, mg/dL | 4.2 (3.1,5.4) | 4.6 (3.4,6.1) | <.001^a)^ |
| Triglycerides, mg/dL | 108.0 (77.0,160.0) | 108.0 (75.0,159.0) | .56 |
| Total cholesterol, mg/dL | 153.0 (125.0,184.0) | 138.0 (104.0,174.0) | <.001^a)^ |
| Calcium, mg/dL | 8.95±0.64 | 8.61±0.99 | <.001^a)^ |

Table S10 (continuation). Basic Statistics for Acute Kidney Injury in the External Cohort

| **Features** | **Non-AKI (*n* = 36,615)** | **AKI (*n* = 2,898)** | ***P*-value** |
| --- | --- | --- | --- |
| Phosphorus, mg/dL | 3.3 (2.8,3.8) | 3.3 (2.7,3.9) | .20 |
| Sodium, mmol/L | 139.99±3.44 | 138.97±7.64 | <.001^a)^ |
| Potassium, mmol/L | 3.99±0.48 | 4.12±0.79 | <.001^a)^ |
| Chloride, mmol/L | 102.37±3.92 | 101.66±8.19 | <.001^a)^ |
| Urine SG | 1.02±0.01 | 1.02±0.01 | <.001^a)^ |
| aPTT, sec | 31.25±8.62 | 34.42±14.84 | <.001^a)^ |
| LDH, U/L | 216.0 (179.0,272.0) | 263.0 (205.0,385.5) | <.001^a)^ |
| C-reactive protein, mg/L | 5.9 (1.7,17.6) | 44.5 (10.0,107.6) | <.001^a)^ |
| pH | 7.4 (7.4,7.5) | 7.4 (7.4,7.5) | <.001^a)^ |
| pCO2, mmHg | 38.2 (34.1,42.4) | 36.1 (30.6,42.0) | <.001^a)^ |
| pO2, mmHg | 83.0 (70.5,100.7) | 86.0 (69.7,111.8) | <.001^a)^ |
| Total CO2, mmol/L | 25.1 (23.2,27.1) | 22.7 (19.3,25.6) | <.001^a)^ |
| Pro-BNP, pg/mL | 175.7 (65.6,539.4) | 538.2 (177.3,2148.5) | <.001^a)^ |
| Nephrotoxic antibiotics† | 1,336 (3.65) | 497 (17.15) | <.001^a)^ |
| NSAIDs† | 10,663 (29.12) | 920 (31.75) | .003^a)^ |
| CHEMOs† | 1,293 (3.53) | 114 (3.93) | .28 |
| Contrast-enhanced CT† | 8,935 (24.4) | 1,036 (35.75) | <.001^a)^ |
| general anesthesia† | 3,381 (9.23) | 466 (16.08) | <.001^a)^ |

Data are presented as mean ± standard deviation, median (interquartile range), or count (%) as appropriate. These features were checked for exposure within one week. ^a)^*P* <0.05. AKI, acute kidney injury; BP, blood pressure; WBC, white blood cell; BUN, blood urea nitrogen; Cr, creatinine; eGFR, estimated glomerular filtration rate; ALT, alanine aminotransferase; ALP, alkaline phosphatase; aPTT, activated partial thromboplastin time; LDH, lactate dehydrogenase; SG, specific gravity; BNP, brain natriuretic peptide; BST, blood sugar test; NSAIDs, non-steroidal anti-inflammatory drugs; CHEMOs, cytotoxic chemotherapeutic agents, CT, computed tomography; pCO2, partial pressure of carbon dioxide; pO2, partial pressure of oxygen. ^a)^ indicate statistically significant.

Table S11. Hyperparameter Tuning Results of the Early Prediction Model for Acute Kidney Injury

| **Model** | | **Parameter** | | | **Range** | |
| --- | --- | --- | --- | --- | --- | --- |
| Logistic Regression | C | | 0.01 | 0.001, 0.01, 0.1, 1.0, 10.0 | |  |
| Random Forest | max_depth | | None | None, 5, 6, 7, 8, 9, 10, 11, 12 | |  |
|  | n_estimators | | 300 | 50, 100, 500, 1000, 2000, 3000 | |  |
|  | min_samples_split | | 2 | 1, 2, 3, 4, 5 | |  |
|  | min_samples_leaf | | 2 | 1, 2, 3, 4, 5 | |  |
| eXtreme Gradient Boosting | max_depth | | 5 | 3, 4, 5, 6, 7, 8, 9, 10, 11, 12 | |  |
|  | learning_rate | | 0.3 | 0.001, 0.003, 0.01, 0.03, 0.05, 0.1, 0.15, 0.2, 0.25, 0.3 | |  |
|  | subsample | | 1 | 0.7, 0.8, 0.9, 1 | |  |
|  | colsample_bytree | | 1 | 0.7, 0.8, 0.9, 1 | |  |
|  | boosting | | gbtree | gbtree, dart | |  |
| Light Gradient Boosting | max_depth | | 8 | -1, 5, 6, 7, 8, 9, 10, 11, 12 | |  |
|  | learning_rate | | 0.1 | 0.001, 0.003, 0.01, 0.03, 0.05, 0.1, 0.15, 0.2, 0.25, 0.3 | |  |
|  | subsample | | 1 | 0.7, 0.8, 0.9, 1 | |  |
|  | colsample_bytree | | 1 | 0.7, 0.8, 0.9, 1 | |  |
|  | boosting | | dart | rf, gbdt, dart, goss | |  |
| Categorical Boosting | depth | | 12 | 3, 4, 5, 6, 7, 8, 9, 10, 11, 12 | |  |
|  | learning_rate | | 0.03 | 0.001, 0.003, 0.009, 0.01, 0.03, 0.05, 0.1, 0.15, 0.2, 0.25, 0.3 | |  |
|  | subsample | | 1 | 0.7, 0.8, 0.9, 1 | |  |
|  | grow_policy | | Lossguide | SymmetricTree, Depthwise, Lossguide | |  |
|  | l2_leaf_reg | | 5 | 1, 2, 3, 5, 10 | |  |

Figure S7. Calibration Plot of the Early Prediction Model for Acute Kidney Injury


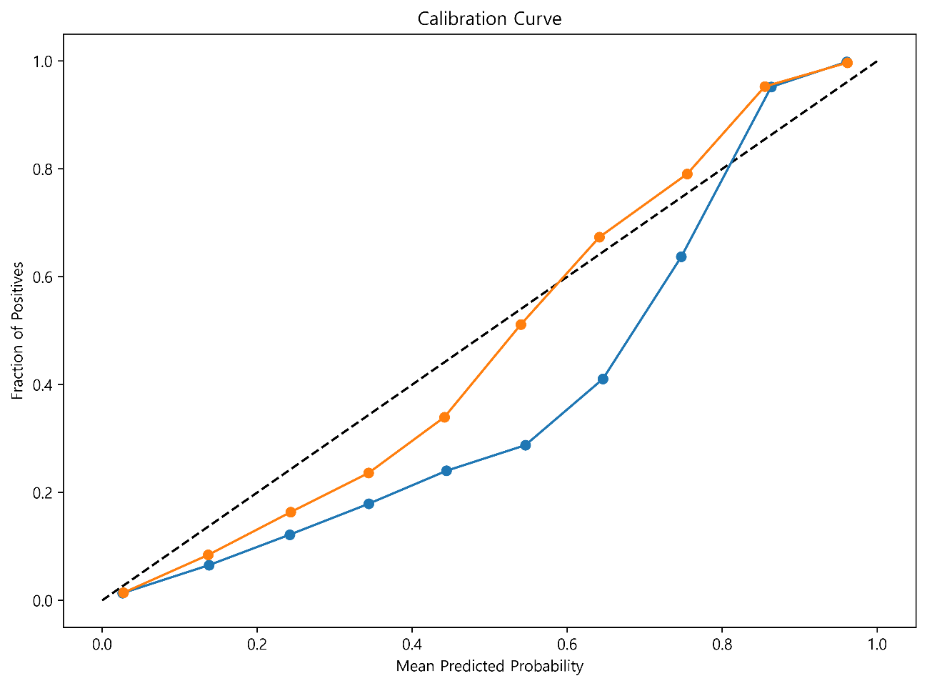


The blue and orange lines represent internal and external data, respectively.

Calibration plots are used to assess the agreement between predicted probabilities and observed outcomes. These plots compare the predicted risk of AKI or AKD with the actual observed incidence across different risk strata. A perfectly calibrated model exhibits a plot in which the predicted probabilities align closely with the 45-degree line. Calibration is evaluated for both internal and external validation cohorts to ensure that the model maintained its predictive performance across different patient populations and settings. AKI, acute kidney injury; AKD, acute kidney disease

Figure S8. Probability Test Results of the Early Prediction Model for Acute Kidney Injury


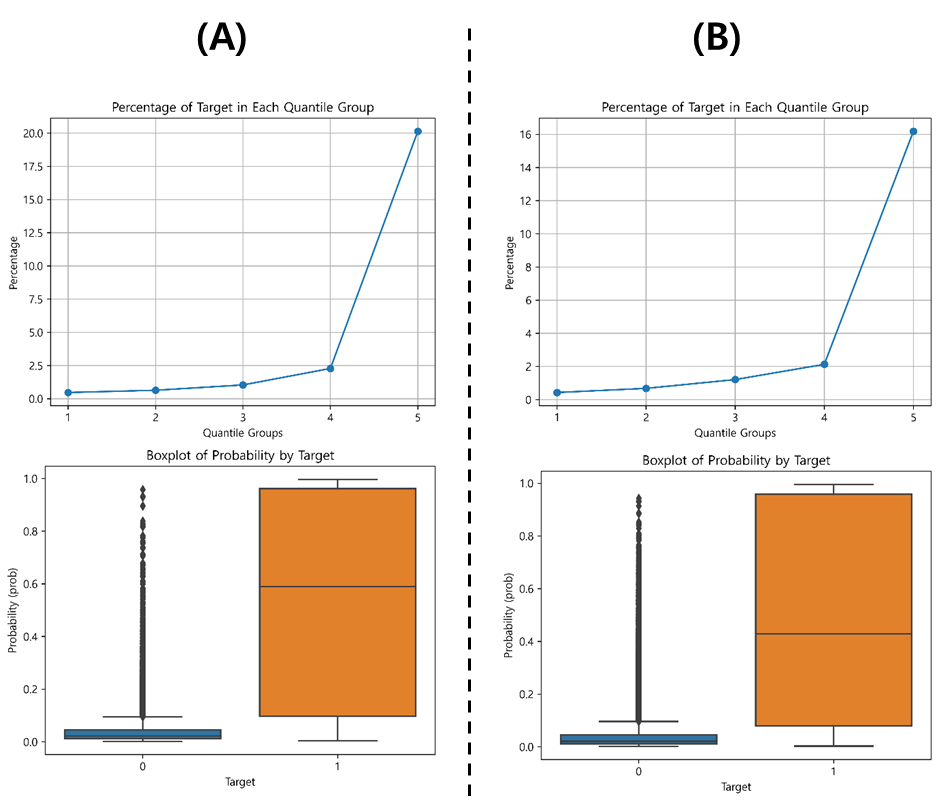


(A) and (B) Represent the internal and external validation results, respectively.

(A) and (B) divide the model's probabilities into five groups and show the proportion of actual events occurring within each patient group. The box plot below illustrates the probabilities derived by the model for Groups 0 and 1. Observing that the Q1 of the group where the event occurred is above the Q3+InterQuartile Range of the group where the event did not occur suggests a high reliability of the probabilities.
