# Supplementary material for: Machine Learning to Assist in Managing Acute Kidney Injury in General Wards: Multicenter Retrospective Study
Source: J Med Internet Res. 2025 Mar 18;27:e66568. doi: 10.2196/66568 (PMC11962325; doi:10.2196/66568)
Supplement: Multimedia Appendix 6 [file jmir_v27i1e66568_app6.docx]

Figure S9. Recovery Patterns of Acute Kidney Injury by Cohort


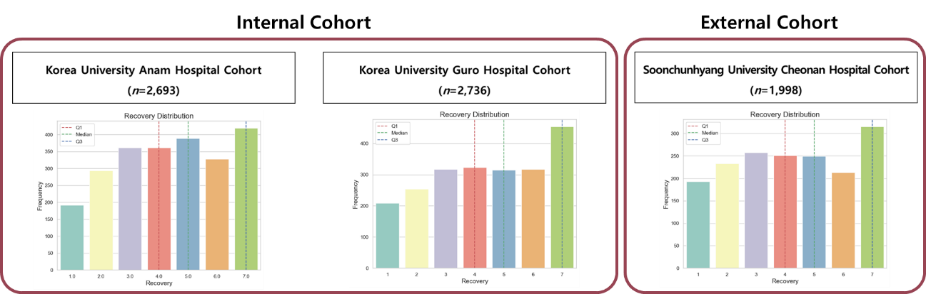


Table S12. Basic Statistics for Acute Kidney Disease in the Internal Cohort

| **Features** | **Non-AKD (*n* = 4,533)** | **AKD (*n* = 896)** | ***P*-value** |
| --- | --- | --- | --- |
| Age, year | 64.99±14.85 | 64.23±14.33 | .16 |
| Male | 1,481 (32.67) | 287 (32.03) | .74 |
| Systolic BP, mmHg | 109.05±36.97 | 107.84±39.39 | .38 |
| Diastolic BP, mmHg | 70.79±13.12 | 71.96±13.06 | .02^a)^ |
| Heart rate, bpm | 90.64±19.43 | 93.23±19.38 | <.001^a)^ |
| Respiratory rate, bpm | 20.0 (18.0,20.0) | 20.0 (18.0,20.0) | .75 |
| Body temperature, °C | 36.99±0.63 | 36.86±0.61 | <.001^a)^ |
| Hemoglobin, g/dL | 10.53±2.23 | 9.90±1.96 | <.001^a)^ |
| WBC count, 10³/μL | 12.86±10.02 | 12.38±9.21 | .18 |
| Platelet, 10³/μL | 188.83±120.38 | 195.15±136.90 | .16 |
| Albumin, g/dL | 3.13±0.58 | 2.86±0.53 | <.001^a)^ |
| BUN, mg/dL | 28.3 (20.4,38.0) | 30.0 (20.7,42.7) | <.001^a)^ |
| Serum Cr, mg/dL | 1.2 (1.0,1.4) | 1.2 (1.0,1.7) | .02^a)^ |
| BUN/Cr ratio | 25.34±12.19 | 26.15±14.39 | .08 |
| eGFR, mL/min | 62.97±20.66 | 60.74±24.79 | .004^a)^ |
| Glucose, mg/dL | 138.0 (110.0,186.0) | 135.0 (109.0,178.0) | .049^a)^ |
| Total bilirubin, mg/dL | 0.9 (0.6,1.6) | 0.8 (0.5,2.0) | .78 |
| ALP, IU/L | 86.0 (62.0,129.0) | 101.0 (72.0,177.0) | <.001^a)^ |
| ALT, IU/L | 25.0 (15.0,48.0) | 28.0 (15.0,54.2) | .04^a)^ |
| BST, mg/dL | 155.0 (123.0,199.2) | 146.0 (120.0,193.0) | .005^a)^ |
| Uric acid, mg/dL | 4.8 (3.5,6.4) | 4.2 (2.8,5.8) | <.001^a)^ |
| Triglycerides, mg/dL | 101.0 (70.0,151.0) | 100.5 (68.2,151.8) | .54 |
| Total cholesterol, mg/dL | 126.0 (96.0,162.0) | 114.0 (87.0,147.0) | <.001^a)^ |
| Calcium, mg/dL | 8.31±0.94 | 8.15±0.91 | <.001^a)^ |

Table S12 (continuation). Basic Statistics for Acute Kidney Disease in the Internal Cohort

| **Features** | **Non-AKD (*n* = 4,533)** | **AKD (*n* = 896)** | ***P*-value** |
| --- | --- | --- | --- |
| Phosphorus, mg/dL | 3.5 (2.9,4.3) | 3.5 (2.9,4.4) | .26 |
| Sodium, mmol/L | 137.72±6.59 | 137.43±8.39 | .25 |
| Potassium, mmol/L | 4.08±0.66 | 4.02±0.72 | .02^a)^ |
| Chloride, mmol/L | 103.82±6.71 | 103.16±8.15 | .009^a)^ |
| Urine SG | 1.02±0.01 | 1.02±0.01 | <.001^a)^ |
| aPTT, sec | 42.20±15.77 | 44.39±17.19 | <.001^a)^ |
| LDH, U/L | 523.0 (397.0,755.0) | 539.0 (412.0,857.0) | .06 |
| C-reactive protein, mg/L | 46.9 (10.6,128.7) | 71.2 (29.4,148.4) | <.001^a)^ |
| pH | 7.4 (7.4,7.5) | 7.4 (7.4,7.5) | .36 |
| pCO2, mmHg | 33.6 (28.7,38.5) | 34.3 (29.2,41.0) | .006^a)^ |
| pO2, mmHg | 94.5 (79.0,126.5) | 91.0 (77.0,125.8) | .15 |
| Total CO2, mmol/L | 23.0 (20.0,26.0) | 22.3 (19.7,26.0) | .42 |
| Pro-BNP, pg/mL | 663.9 (192.4,2230.0) | 576.2 (176.3,1782.5) | .07 |
| Nephrotoxic antibiotics† | 1,037 (22.88) | 447 (49.89) | <.001^a)^ |
| NSAIDs† | 1,604 (35.38) | 286 (31.92) | .05 |
| CHEMOs† | 427 (9.42) | 84 (9.38) | 1.00 |
| Contrast-enhanced CT† | 2,143 (47.28) | 359 (40.07) | <.001^a)^ |
| general anesthesia† | 1,581 (34.88) | 181 (20.2) | <.001^a)^ |

Data are presented as mean ± standard deviation, median (interquartile range), or count (%) as appropriate. These features were checked for exposure within one week. ^a)^*P* <0.05. AKI, acute kidney injury; BP, blood pressure; WBC, white blood cell; BUN, blood urea nitrogen; Cr, creatinine; eGFR, estimated glomerular filtration rate; ALT, alanine aminotransferase; ALP, alkaline phosphatase; aPTT, activated partial thromboplastin time; LDH, lactate dehydrogenase; SG, specific gravity; BNP, brain natriuretic peptide; BST, blood sugar test; NSAIDs, non-steroidal anti-inflammatory drugs; CHEMOs, cytotoxic chemotherapeutic agents, CT, computed tomography; pCO2, partial pressure of carbon dioxide; pO2, partial pressure of oxygen. ^a)^ indicate statistically significant.

Table S13. Basic statistics for Acute Kidney Disease in the External Cohort

| **Features** | **Non-AKD (*n* = 1,711)** | **AKD (*n* = 287)** | ***P*-value** |
| --- | --- | --- | --- |
| Age, year | 65.60±15.56 | 63.12±14.94 | .01^a)^ |
| Male | 563 (32.9) | 102 (35.54) | .42 |
| Systolic BP, mmHg | 131.42±21.30 | 129.58±20.65 | .17 |
| Diastolic BP, mmHg | 80.25±12.07 | 79.09±11.87 | .13 |
| Heart rate, bpm | 101.01±24.75 | 101.52±22.94 | .74 |
| Respiratory rate, bpm | 20.0 (18.0,25.0) | 20.0 (18.0,25.0) | .42 |
| Body temperature, °C | 37.33±0.75 | 37.27±0.70 | .19 |
| Hemoglobin, g/dL | 10.93±2.23 | 10.63±2.05 | .03^a)^ |
| WBC count, 10³/μL | 12.13±8.56 | 12.21±10.23 | .88 |
| Platelet, 10³/μL | 209.72±116.79 | 210.99±128.46 | .87 |
| Albumin, g/dL | 3.10±0.67 | 2.82±0.68 | <.001^a)^ |
| BUN, mg/dL | 22.4 (16.1,31.9) | 24.5 (17.0,36.8) | .006^a)^ |
| Serum Cr, mg/dL | 1.1 (1.0,1.4) | 1.2 (1.0,1.8) | <.001^a)^ |
| BUN/Cr ratio | 21.59±11.45 | 20.93±11.40 | .37 |
| eGFR, mL/min | 65.25±20.89 | 58.63±25.94 | <.001^a)^ |
| Glucose, mg/dL | 129.0 (105.0,170.0) | 120.0 (96.0,159.0) | <.001^a)^ |
| Total bilirubin, mg/dL | 0.7 (0.4,1.2) | 0.6 (0.4,1.4) | .97 |
| ALP, IU/L | 78.0 (57.0,122.5) | 94.0 (67.5,157.0) | <.001^a)^ |
| ALT, IU/L | 22.0 (13.0,40.0) | 23.0 (13.0,47.5) | .39 |
| BST, mg/dL | 153.9 (127.3,198.7) | 144.2 (120.0,185.6) | .007^a)^ |
| Uric acid, mg/dL | 4.7 (3.5,6.1) | 4.4 (3.1,6.1) | .05 |
| Triglycerides, mg/dL | 105.0 (73.0,156.5) | 110.0 (80.0,165.0) | .049^a)^ |
| Total cholesterol, mg/dL | 139.0 (107.0,175.0) | 136.0 (103.0,177.0) | .42 |
| Calcium, mg/dL | 8.66±0.86 | 8.50±1.13 | .006^a)^ |

Table S13 (continuation). Basic Statistics for Acute Kidney Disease in the External Cohort

| **Features** | **Non-AKD (*n* = 1,711)** | **AKD (*n* = 287)** | ***P*-value** |
| --- | --- | --- | --- |
| Phosphorus, mg/dL | 3.3 (2.7,3.9) | 3.3 (2.7,3.9) | .81 |
| Sodium, mmol/L | 138.41±6.19 | 138.33±8.22 | .85 |
| Potassium, mmol/L | 4.06±0.69 | 4.00±0.77 | .18 |
| Chloride, mmol/L | 101.29±6.74 | 100.82±8.73 | .30 |
| Urine SG | 1.02±0.01 | 1.02±0.01 | <.001^a)^ |
| aPTT, sec | 33.15±12.22 | 34.48±10.06 | .09 |
| LDH, U/L | 246.0 (196.0,329.0) | 265.5 (201.0,406.2) | .01^a)^ |
| C-reactive protein, mg/L | 38.0 (8.2,96.0) | 55.2 (16.5,115.2) | .001^a)^ |
| pH | 7.4 (7.4,7.5) | 7.4 (7.4,7.5) | .008^a)^ |
| pCO2, mmHg | 36.0 (30.8,40.9) | 35.5 (30.5,40.2) | .37 |
| pO2, mmHg | 88.4 (72.0,115.4) | 87.6 (71.2,109.5) | .51 |
| Total CO2, mmol/L | 22.7 (19.8,25.4) | 23.4 (19.4,26.8) | .06 |
| Pro-BNP, pg/mL | 483.8 (152.6,2060.5) | 429.6 (167.7,1409.5) | .51 |
| Nephrotoxic antibiotics† | 213 (12.45) | 107 (37.28) | <.001^a)^ |
| NSAIDs† | 591 (34.54) | 82 (28.57) | .06 |
| CHEMOs† | 68 (3.97) | 16 (5.57) | .28 |
| Contrast-enhanced CT† | 653 (38.16) | 93 (32.4) | .07 |
| general anesthesia† | 360 (21.04) | 24 (8.36) | <.001^a)^ |

Data are presented as mean ± standard deviation, median (interquartile range), or count (%) as appropriate. These features were checked for exposure within one week. ^a)^*P* <0.05. AKI, acute kidney injury; BP, blood pressure; WBC, white blood cell; BUN, blood urea nitrogen; Cr, creatinine; eGFR, estimated glomerular filtration rate; ALT, alanine aminotransferase; ALP, alkaline phosphatase; aPTT, activated partial thromboplastin time; LDH, lactate dehydrogenase; SG, specific gravity; BNP, brain natriuretic peptide; BST, blood sugar test; NSAIDs, non-steroidal anti-inflammatory drugs; CHEMOs, cytotoxic chemotherapeutic agents, CT, computed tomography; pCO2, partial pressure of carbon dioxide; pO2, partial pressure of oxygen. ^a)^ indicate statistically significant.

Table S14. Hyperparameter Tuning Results of the Early Prediction Model for Acute Kidney Disease

| **Model** | | **Parameter** | | | **Range** | |
| --- | --- | --- | --- | --- | --- | --- |
| Logistic Regression | C | | 0.1 | 0.001, 0.01, 0.1, 1.0, 10.0 | |  |
| Random Forest | max_depth | | 9 | None, 5, 6, 7, 8, 9, 10, 11, 12 | |  |
|  | n_estimators | | 300 | 50, 100, 500, 1000, 2000, 3000 | |  |
|  | min_samples_split | | 1 | 1, 2, 3, 4, 5 | |  |
|  | min_samples_leaf | | 1 | 1, 2, 3, 4, 5 | |  |
| eXtreme Gradient Boosting | max_depth | | 9 | 3, 4, 5, 6, 7, 8, 9, 10, 11, 12 | |  |
|  | learning_rate | | 0.3 | 0.001, 0.003, 0.01, 0.03, 0.05, 0.1, 0.15, 0.2, 0.25, 0.3 | |  |
|  | subsample | | 1 | 0.7, 0.8, 0.9, 1 | |  |
|  | colsample_bytree | | 1 | 0.7, 0.8, 0.9, 1 | |  |
|  | boosting | | gbtree | gbtree, dart | |  |
| Light Gradient Boosting | max_depth | | 10 | -1, 5, 6, 7, 8, 9, 10, 11, 12 | |  |
|  | learning_rate | | 0.1 | 0.001, 0.003, 0.01, 0.03, 0.05, 0.1, 0.15, 0.2, 0.25, 0.3 | |  |
|  | subsample | | 1 | 0.7, 0.8, 0.9, 1 | |  |
|  | colsample_bytree | | 1 | 0.7, 0.8, 0.9, 1 | |  |
|  | boosting | | dart | rf, gbdt, dart, goss | |  |
| Categorical Boosting | depth | | 11 | 3, 4, 5, 6, 7, 8, 9, 10, 11, 12 | |  |
|  | learning_rate | | 0.03 | 0.001, 0.003, 0.009, 0.01, 0.03, 0.05, 0.1, 0.15, 0.2, 0.25, 0.3 | |  |
|  | subsample | | 1 | 0.7, 0.8, 0.9, 1 | |  |
|  | grow_policy | | Lossguide | SymmetricTree, Depthwise, Lossguide | |  |
|  | l2_leaf_reg | | 5 | 1, 2, 3, 5, 10 | |  |

Figure S10. Calibration Plot of the Early Prediction Model for Acute Kidney Disease


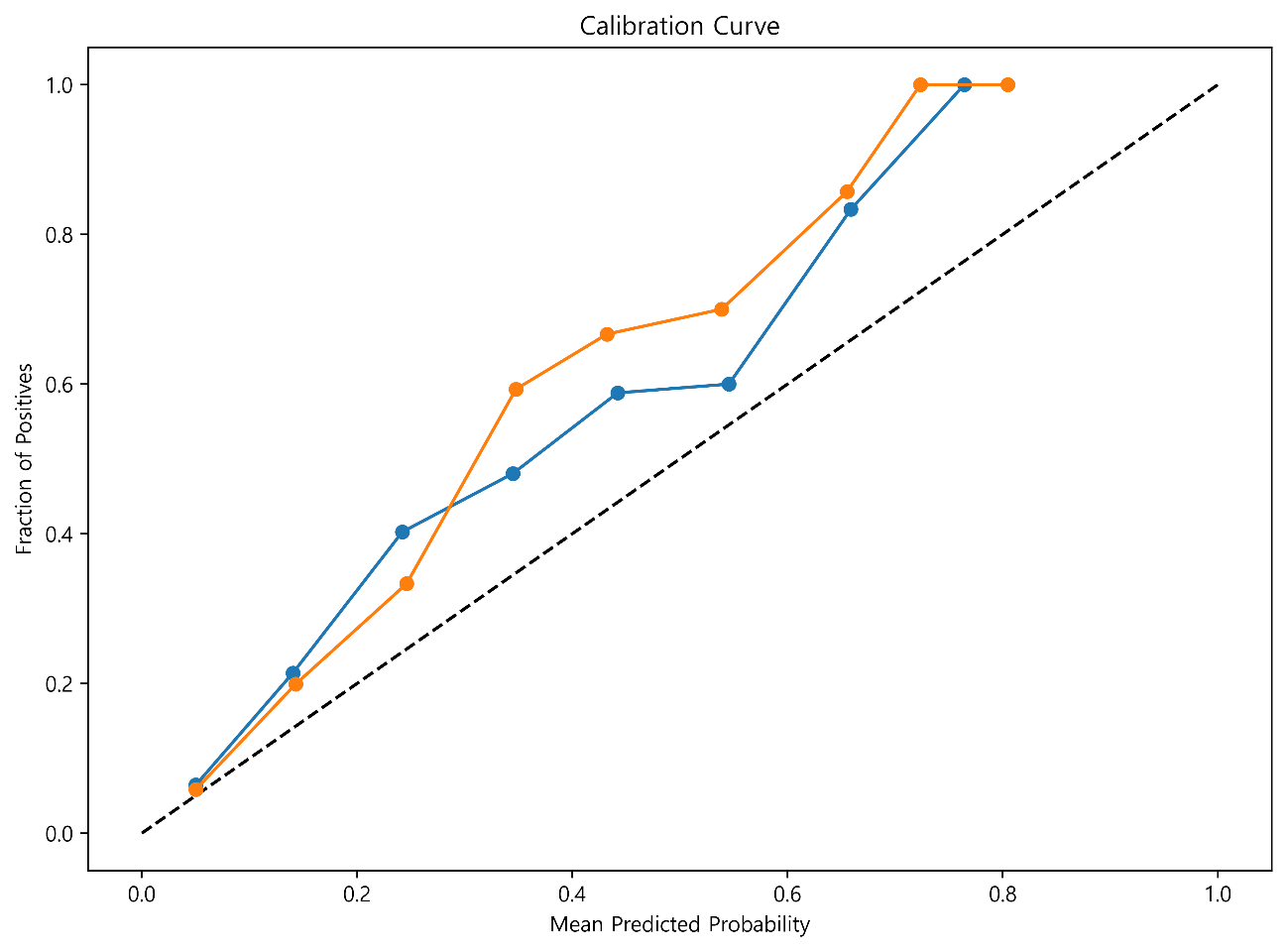


The blue and orange lines represent internal and external data, respectively.

Calibration plots are used to assess the agreement between predicted probabilities and observed outcomes. These plots compare the predicted risk of AKI or AKD with the actual observed incidence across different risk strata. A perfectly calibrated model exhibits a plot in which the predicted probabilities align closely with the 45-degree line. Calibration is evaluated for both internal and external validation cohorts to ensure that the model maintained its predictive performance across different patient populations and settings.

Figure S11. Probability Test Results of the Early Prediction Model for Acute Kidney Disease


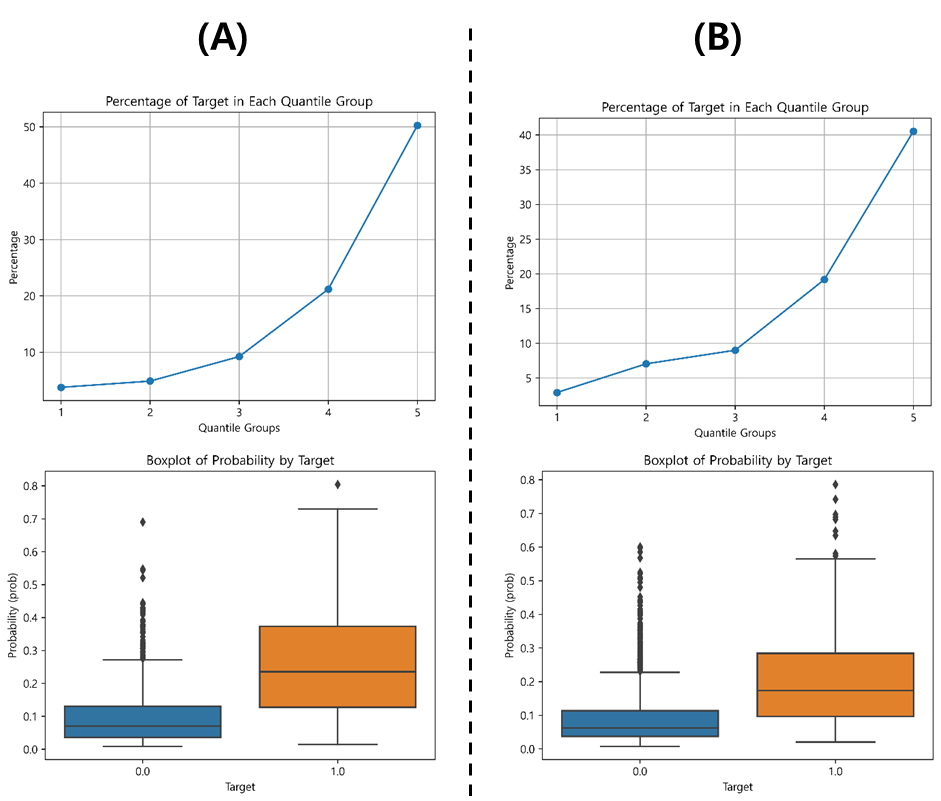


(A) and (B) represent the internal and (external validation results, respectively.

(A) and (B) divide the model's probabilities into five groups and show the proportion of actual events occurring within each patient group. The box plot below illustrates the probabilities derived by the model for Groups 0 and 1.
