# Supplementary material for: Machine Learning to Assist in Managing Acute Kidney Injury in General Wards: Multicenter Retrospective Study
Source: J Med Internet Res. 2025 Mar 18;27:e66568. doi: 10.2196/66568 (PMC11962325; doi:10.2196/66568)
Supplement: Multimedia Appendix 7 [file jmir_v27i1e66568_app7.docx]

Figure S13. Framework to Assist in Managing Acute Kidney Injury


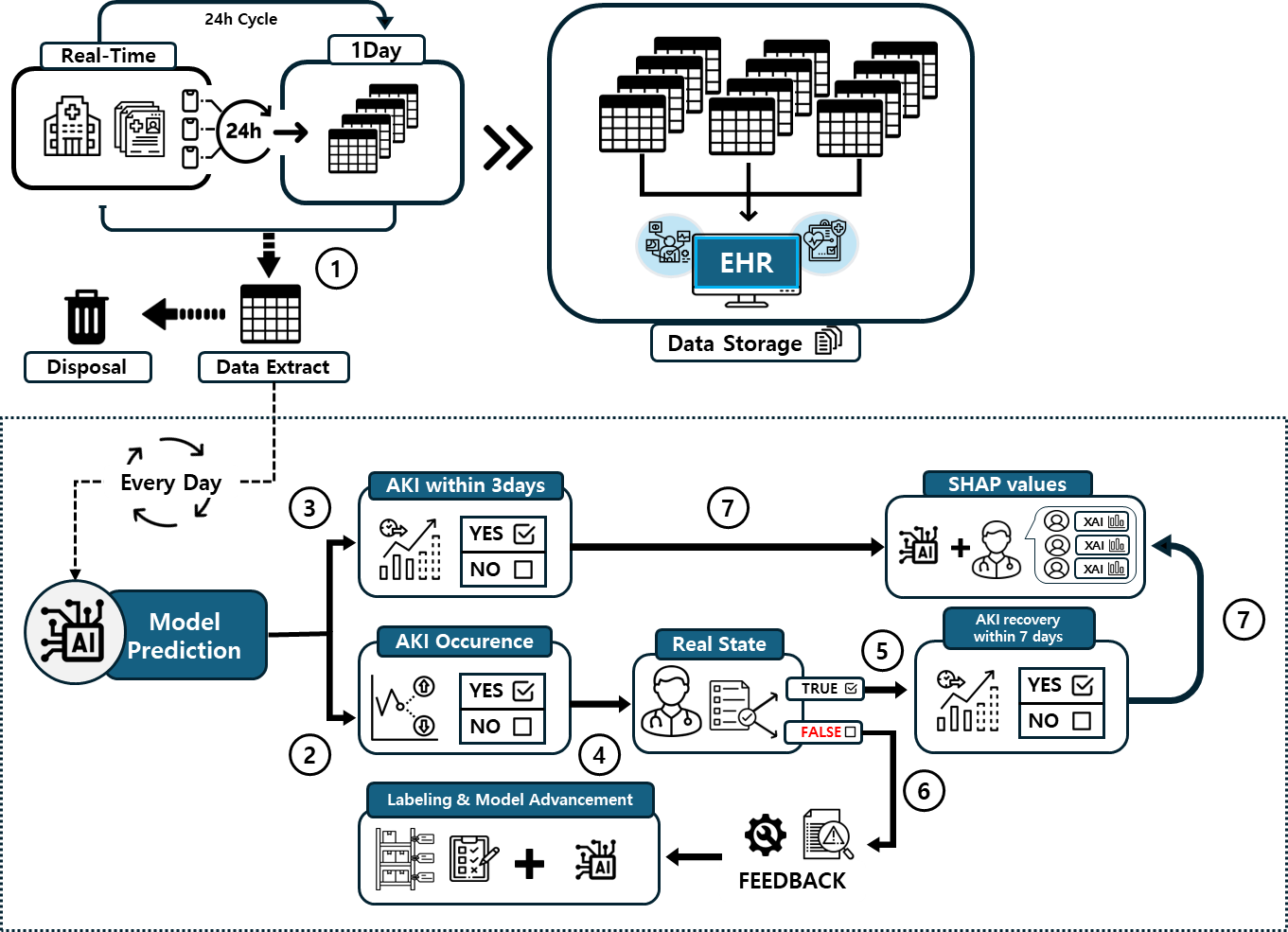


AI, artificial intelligence; EHR, electronic health records; AKI, acute kidney injury; SHAP, SHapley Additive exPlanations values; XAI, eXplainable artificial intelligence; The numbers indicated in the figure represent the flow of the framework. Detailed information is provided in eContent 5.

Two CAT models developed for the early prediction of AKI and AKD have been integrated into clinical practice. The designed framework is illustrated in eFigure 13. A cut-off adjustment was made to maintain a recall of 0.7. eFigure 14 illustrates the performance variations according to the cutoff values for each model.

1. Data from newly measured patients for the day were copied at a fixed time (e.g., 2 AM) and processed using the same preprocessing pipeline as in the model development.
2. Patients suspected of experiencing AKI based on predefined criteria were identified.
3. Simultaneously, machine learning models listed patients who were not currently experiencing AKI but were expected to develop AKI within 3 days.
4. Physicians determined the actual occurrence of AKI for patients suspected of having AKI.
5. If AKI occurred, machine learning models predicted recovery within 7 days.
6. Data from patients identified as having AKI but not actually developing AKI were used to supplement the AKI labeling criteria using retrospective creatinine data.
7. For patients predicted to develop AKI within 3 days or those who did not recover early after AKI, SHAP values were provided for each patient.

Figure S14. Comparison of Model Performance Based on Cutoff Values


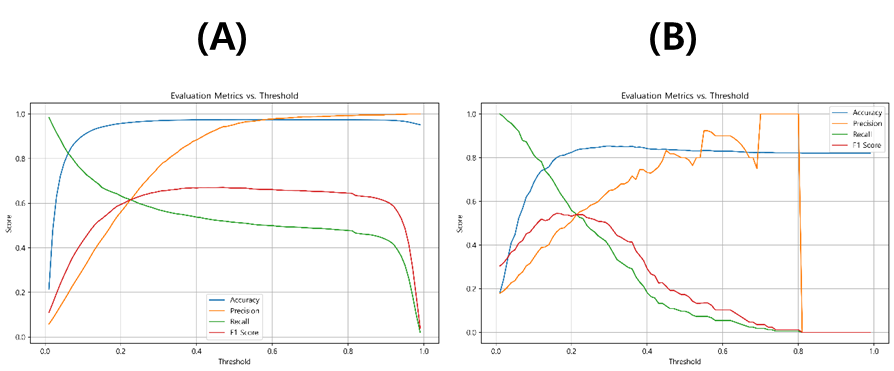


(A) Results of the early prediction model for acute kidney injury, and (B) results of the early prediction model for acute kidney disease.

Figure S15. Simulation Results of the Acute Kidney Injury Management Framework Using External Validation Data


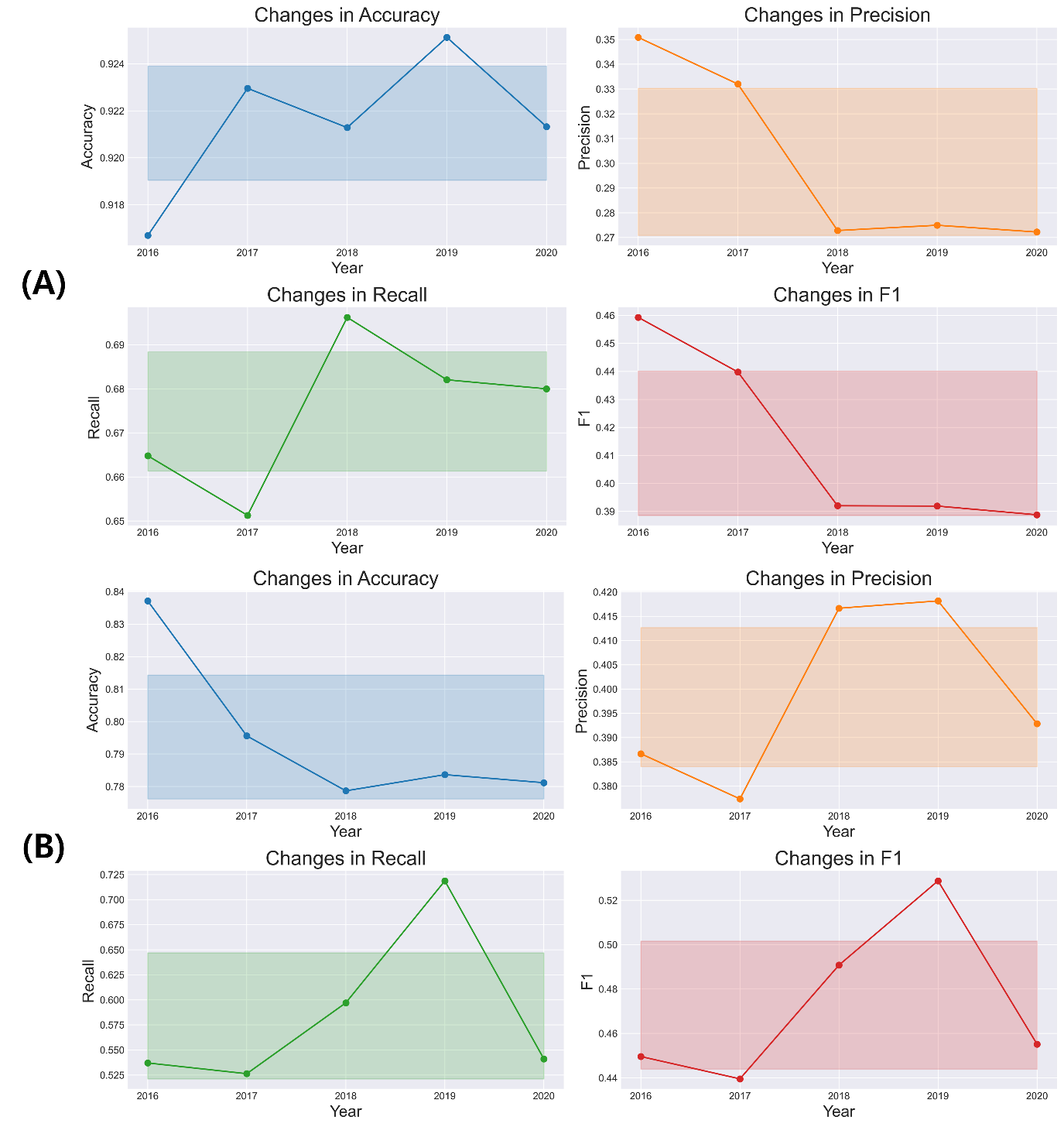


(A) Results of the early prediction model for acute kidney injury, and (B) results of the early prediction model for acute kidney disease. Blue, orange, green, and red indicate the accuracy, precision, recall, and F1, respectively. In each graph, the vertical line represents the mean and the shaded area represents the 95% confidence interval.
